# WingAnalogy: a computer vision-based tool for automated insect wing asymmetry and morphometry analysis

**DOI:** 10.1038/s41598-024-73411-x

**Published:** 2024-09-27

**Authors:** Shahab Eshghi, Hamed Rajabi, Natalia Matushkina, Lisa Claußen, Johannes Poser, Thies H. Büscher, Stanislav N. Gorb

**Affiliations:** 1 Department of Functional Morphology and Biomechanics, Zoological Institute, Kiel University, 24118 Kiel, Germany; 2 Division of Mechanical Engineering and Design, School of Engineering, London South Bank University, London, UK; 3 Mechanical Intelligence Research Group, School of Engineering, London South Bank University, London, UK; 4 Institute of Biology and Medicine, Taras Shevchenko National University of Kyiv, Kyiv, Ukraine

**Keywords:** Entomology, Fluctuating asymmetry, Analogy, PSO, Procrustes superimposition, Software, Imaging

## Abstract

WingAnalogy is a computer tool for automated insect wing morphology and asymmetry analysis. It facilitates project management, enabling users to import pairs of wing images obtained from individual insects, such as left and right, fore- and hindwings. WingAnalogy employs image processing and computer vision to segment wing structures and extract cell boundaries, and junctions. It quantifies essential metrics encompassing cell and wing characteristics, including area, length, width, circularity, and centroid positions. It enables users to scale and superimpose wing images utilizing Particle Swarm Optimization (PSO). WingAnalogy computes regression, Normalized Root Mean Square Error (NRMSE), various cell-based parameters, and distances between cell centroids and junctions. The software generates informative visualizations, aiding researchers in comprehending and interpreting asymmetry patterns. WingAnalogy allows for dividing wings into up to five distinct wing cell sets, facilitating localized comparisons. The software excels in report generation, providing detailed asymmetry measurements in PDF, CSV, and TXT formats.

## Introduction

With their delicate and intricate wings, insects provide a canvas for scientists and researchers to explore the mysteries of evolution, adaptation, and biodiversity^1^. The study of insect wing morphology has been a cornerstone question of entomology, offering profound insights into biomechanics, animal behavior, physiology, and ecology^2–11^. Researchers have used wing characters, especially venation patterns^12,13^ for decades to reconstruct the phylogeny and evolutionary history of insects and establish diagnostic characters to differentiate species and higher taxonomic levels^14,15^. Additionally, insect wings fascinated researchers for their vital roles in damage prevention^16^, thermoregulation^17^, and communication^18^. Through the examination of wing structures, researchers have revealed insights into the evolutionary adaptations, species diversity, and ecological roles of insects^19–22^. While wing structure holds immense significance, researchers have faced persistent challenges in analyzing complex wing venation that have prevented their quest for precise and comprehensive results. Conventional manual techniques proved to be time-consuming, prone to human mistakes, and frequently fell short of the precision demanded by difficult scientific inquiries^23,24^. The demand for advanced, automated, and user-friendly data-driven solutions became increasingly evident, prompting the development of innovative software tools^25,26^.

Before this study, the authors established their expertise in software development by creating several computer tools for studying and analyzing insect wings^27–30^. WingMesh was designed to generate mesh structures along the boundaries of insect wings, utilizing computer vision techniques with Distmesh2D^27,29,31^. Another software, WingGram, was developed to perform finite element modeling of insect wings. This was achieved using Python scripting in Abaqus, in conjunction with computer vision methodologies^32^. The authors also introduced WingSegment, a software application designed to segment insect wing images into constituent elements, including cells, junctions, and veins^28^. This process involved employing various image processing, computer vision, and graph theory techniques, such as region-growing^33–36^, pathfinding^37^, thinning^38^, and line simplification^39,40^. Furthermore, WingSegment had practical applications for generating 3D printable models of insect wings from their images, accomplished through FreeCAD Python scripting by generating macro files^28^.

While the mentioned software packages are primarily designed for tasks, such as extracting geometric features of insect wings, finite element analysis, or 3D modeling of the wing, they seem to lack specific tools for automated analysis of the insect wings’ asymmetry. However, it is important to note that investigating wing asymmetry in insects is very important, as it provides valuable insights into various areas, including flight biomechanics, evolutionary adaptations, ecological interactions, environmental health monitoring, taxonomic identification, and the potential for applications in biomimetics and materials science^41,42^. It is important to note that the study of insect wing asymmetry has developed significantly with technological advancements. In earlier research, manual cell counting was employed to compare specific wing areas, such as those between the nodus and pterostigma along the coastal margin in damselflies^43^. Subsequent studies expanded their analysis to include wing length measurements alongside manual cell counting in damselflies^44^. For a more in-depth examination of wing three-dimensional structures, micro-CT scanners have been utilized^45^. However, while meticulous, this method is too complex and time-consuming for analyzing many individuals. Another research introduced automated software to count cells in photographed wings, streamlining the process^46^. Moreover, in another study, researchers combined data from various Odonata species to create a predictive model for wing venation, utilizing both scanned and photographed wings^20^. Meanwhile, other researchers adopted landmark-based techniques to compare the overall shape of specific wing areas^22,47–50^.

In this paper, we aim to address the lack of previous methods regarding manual analysis or landmark-based methods in studying the asymmetry of insect wings by introducing a new software, WingAnalogy. Featuring a user-friendly graphical interface, WingAnalogy assists a broader audience, including scientists without programming expertise. The design of WingAnalogy draws upon a diverse array of algorithms encompassing computer vision, image processing, statistics, and metaheuristics, all comprehensively detailed within this paper. We conducted manual measurements using ImageJ to validate the accuracy of WingAnalogy’s results. Case study species were chosen to represent wings of increasing complexity (number of cells and junctions): *Apis mellifera* (Honeybee, Hymenoptera; forewings), *Ischnura elegans* (Blue-tailed damselfly, Odonata: Zygoptera; forewings), *Crocothemis erythraea* (Scarlet dragonfly, Odonata: Anisoptera; forewings), and *Schistocerca gregaria* (Desert locust, Orthoptera; hindwings). In the subsequent sections, we delve into the advancements brought forth by WingAnalogy while also addressing its limitations.

## Methods

WingAnalogy, developed using MATLAB App Designer (Version 2020b), is a software tool that streamlines and simplifies the computation of asymmetry in pairs of insect wings. This tool assesses wing asymmetry based on 2D wing images. The criteria for evaluating wing asymmetry encompass regression and normalized root mean square error calculations involving parameters such as area, length, width, and circularity of corresponding wing cells, the distances between junctions and outlines, and the number of cells and junctions. Upon importing a wing image, the integrated algorithm within WingAnalogy segments the image by extracting the wing outline and cells’ boundaries and identifying the junction locations. Once the image is segmented, users can scale the wing. The subsequent step involves superimposing the imported wings, which are carried out automatically or manually. Subsequently, the embedded functionality in the software measures the extent of asymmetry in the wings. Users can divide the wing into a maximum of five cell sets during this stage, allowing for localized comparisons between different wing cell sets. A noteworthy feature of WingAnalogy is its capability to import multiple wing pairs (forewing, hindwings within a species, or specific pairs of wings in different species), enabling users to comprehensively compare the asymmetry results across all wing pairs and wing pair sets. Upon completing asymmetry calculations, users can generate a TXT and a CSV file containing comprehensive asymmetry data and multiple Figures detailing cell attributes (area, width, length, circularity) and their regression analyses between paired wings. The user can also generate a PDF report of all measurements and analyses.

### Image preparation

**Fig. 1:**
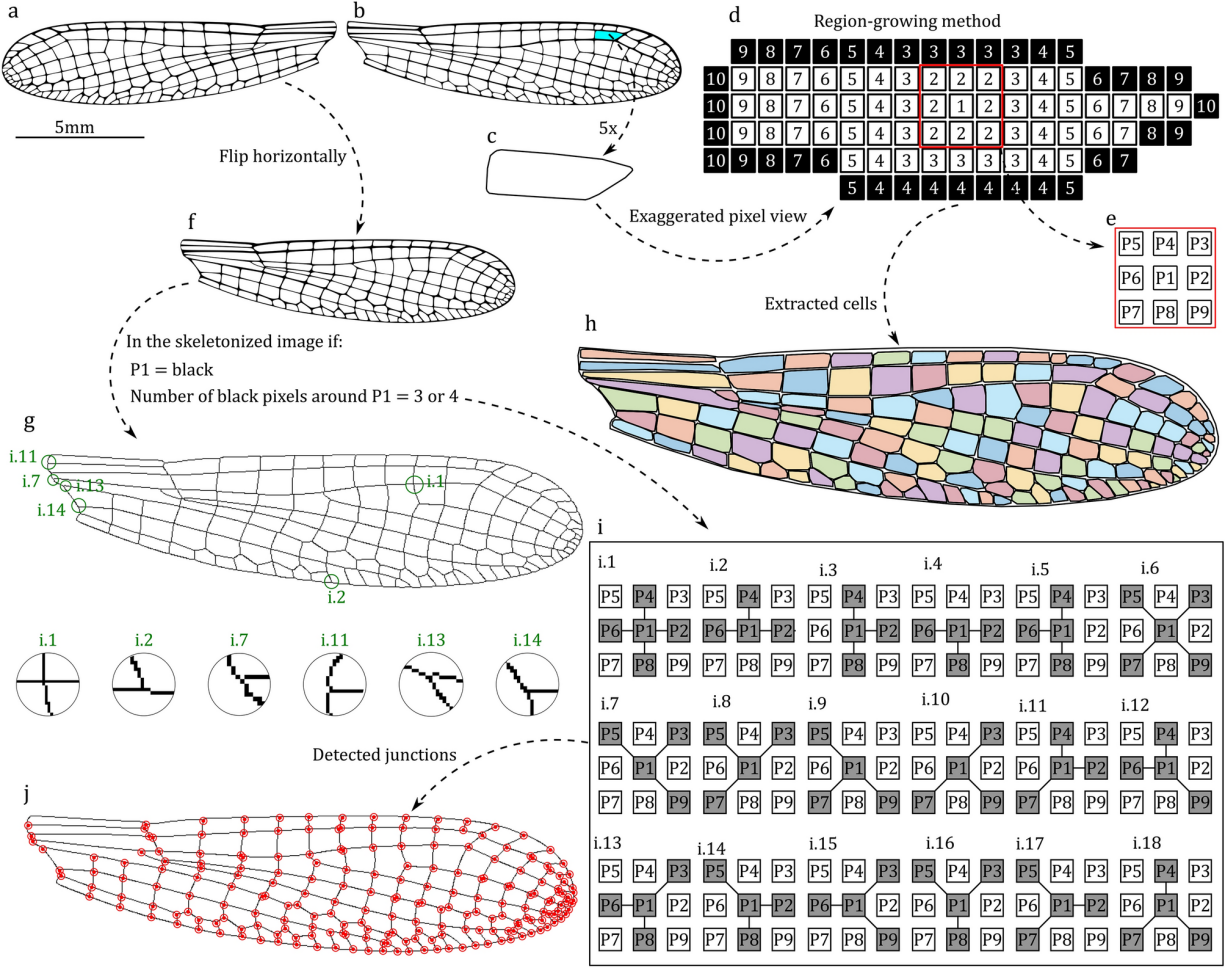
Illustration of the WingAnalogy segmentation process. (**a**) and (**b**) The left and right wings of damselfly wings, respectively. (**c**) A selected cell within the right wing. (**d**) An exaggerated view of the cell is highlighted in (**c**). (**e**) A pixel with its surrounding pixels. (**f**) The mirrored image of the left wing from (**a**). (**g**) The skeletonized image of the wing from (**f**). (**h**) Extracted wing cell boundaries from the wing image in (**b**) using the region growing method. (**i**) The conditions that, if met, classify a pixel as a junction. (**j**) The detected junctions within the wing.

Ensuring optimal image quality is important because it directly influences data extraction accuracy. Additionally, both comparison images must share the same dimensions and resolution. Images can be obtained through a camera or scanner. As demonstrated in Fig. 1a,b, we illustrate the image of the left and right damselfly wing obtained using a high-resolution scanner (Epson Perfection V850 Pro). One crucial guideline in WingAnalogy is to ensure that all images are oriented in the same direction. Therefore, as shown in Fig. 1f, it is required to mirror the left wing to align it with the right wing before conducting wing analysis. Users must ensure that all wing veins and membranes remain distinctly visible after the binarization process by converting the image into a binary representation consisting solely of black and white pixels determined by a threshold value of 0.54. For reference, Fig. 1a and b are suitable examples of an image that aligns with the prerequisites for effective utilization within the WingAnalogy functionality. In addition, Sample Images S1 to S8 in the supplementary files^51^ are images specifically chosen for analysis in this study. Furthermore, Code S1 in the supplementary information^51^ is the code developed to import wing images in WingAnalogy. Video S1 in the supplementary files^51^ demonstrates the adequate reparation of a wing image. While manual preparation can improve results, it is not always required. Video S1 was designed to demonstrate the worst-case scenario, whereas simpler edits are typically adequate.

### Image segmentation

Upon importation, every image undergoes an automated segmentation process formulated according to computer vision principles, and image processing detects all boundaries within the wing. In this approach, a region-growing algorithm is employed to extract cell boundaries^33–36^. Figure 1d provides an exaggerated view of the cell highlighted in Fig. 1c, which is selected from the right wing of the damselfly depicted in Fig. 1b. The region-growing method needs an initial point, such as pixel number 1 in Fig. 1d. As demonstrated in Fig. 1e, a random pixel, like p1, is surrounded by eight neighboring pixels (i.e. P2-P9). Subsequently, the initial pixel 1 examines all surrounding pixels. A white pixel is saved as the front pixel as the method proceeds its search using front pixels. Conversely, if a pixel is black, the algorithm records its coordinates as the boundary of that particular region. During each iteration, the algorithm changes the color of each detected pixel to prevent redundancy. This process persists until all white pixels within the boundary have been examined. At this stage, all boundary pixels are identified. The algorithm then proceeds to another region and repeats the same procedure. As a result, the region-growing method ultimately identifies all cell boundaries within the wing. Figure 1h displays the detected boundaries of the image in Fig. 1b, achieved through the region-growing method. After boundary extraction, the number of detected white pixels inside each region represents the area of that cell. The maximum distance between the boundary of each cell represents the length. Width is measured by dividing the area by the length, and the circularity is measured using Eq. (1):

1 $$\begin{aligned} C = (4\pi \times area)/(perimeter^{2}) \end{aligned}$$

In Eq. (1), the area is the area of the cell, and the perimeter is the perimeter of the cell measured by the region-growing algorithm.

All measurements about wing size and its constituent cells are conducted in pixel units. Consequently, an integrated feature within WingAnalogy enables users to scale all measurements. Figure S4 shows the distribution of cells area, length, width, and circularity and corresponding histograms of the wing in Fig. 1b. Moreover, WingAnalogy employs a thinning algorithm to skeletonize imported images^38^. Figure 1g illustrates the skeletonized version of Fig. 1f. Within the skeletonized image, any black pixel surrounded by 3 or 4 other black pixels (essentially, any pixel meeting one of the 18 conditions outlined in Fig. 1i), is identified as a junction. Figure 1j presents all the detected junctions on the wing. Moreover, Codes S2, S3, and S4 in the supplementary information^51^ are developed for the region-growing method, wing segmentation, and wing junction detection.

### Superimposing

The primary goal of superimposition using the Procrustes method is to eliminate differences in position, orientation, and scale between shapes so that they can be more easily compared and analyzed^52^. Superimposing in the context of comparing two images refers to overlaying or combining two images on top of each other to analyze their differences and similarities visually. In WingAnalogy, we skip scaling between shapes because we assume that the images used are captured at the same size. This is because, during wing comparisons, there might be instances where one wing is larger or smaller, and this size difference could be an essential criterion for comparison^53^. In the WingAnalogy software, this step follows the automatic extraction of geometry and scaling data and serves as the initial phase for comparing insect wings. The superimposing process can be carried out either manually or automatically.

#### Manual superimposing

For manual superimposition, the software incorporates a set of ten buttons illustrated in Fig. S5 that facilitate wing translation and rotation. Notably, during this process, users view the wings within a display panel, observing the extracted wing boundaries rather than the complete wing images. In this process, one wing remains fixed as a reference, while the position of the other wing can be adjusted solely to achieve alignment. It is important to highlight that the software does not limit users to relying exclusively on visual superimposition. A text box integrated into the software displays distances between outline points, cells’ centroids, and junctions. This feature empowers users to quantitatively assess the impact of positional adjustments beyond just visual inspection. As a result, users can determine whether changing the wing’s position effectively minimizes these measured distances.

#### Automated superimposing

The automated superimposition is often more efficient and time-saving than the manual superimposition. The *Auto superimposing* button in Fig. S5 is embedded for this purpose. This approach utilizes Particle Swarm Optimization (PSO) to perform translations and rotations on one wing, minimizing the differences between wing outlines^54^. The PSO algorithm works with a population called a swarm consisting of candidate solutions known as particles. The PSO algorithm is configured with the following parameters:

- Number of variables: 3 (x, y, theta)
- Maximum iterations: 40
- Number of particles: 30
- Inertia weight (w): 1
- Damping coefficient (wDamp): 0.95
- Cognitive parameter (C1): 1.2
- Social parameter (C2): 1.2

**Fig. 2:**
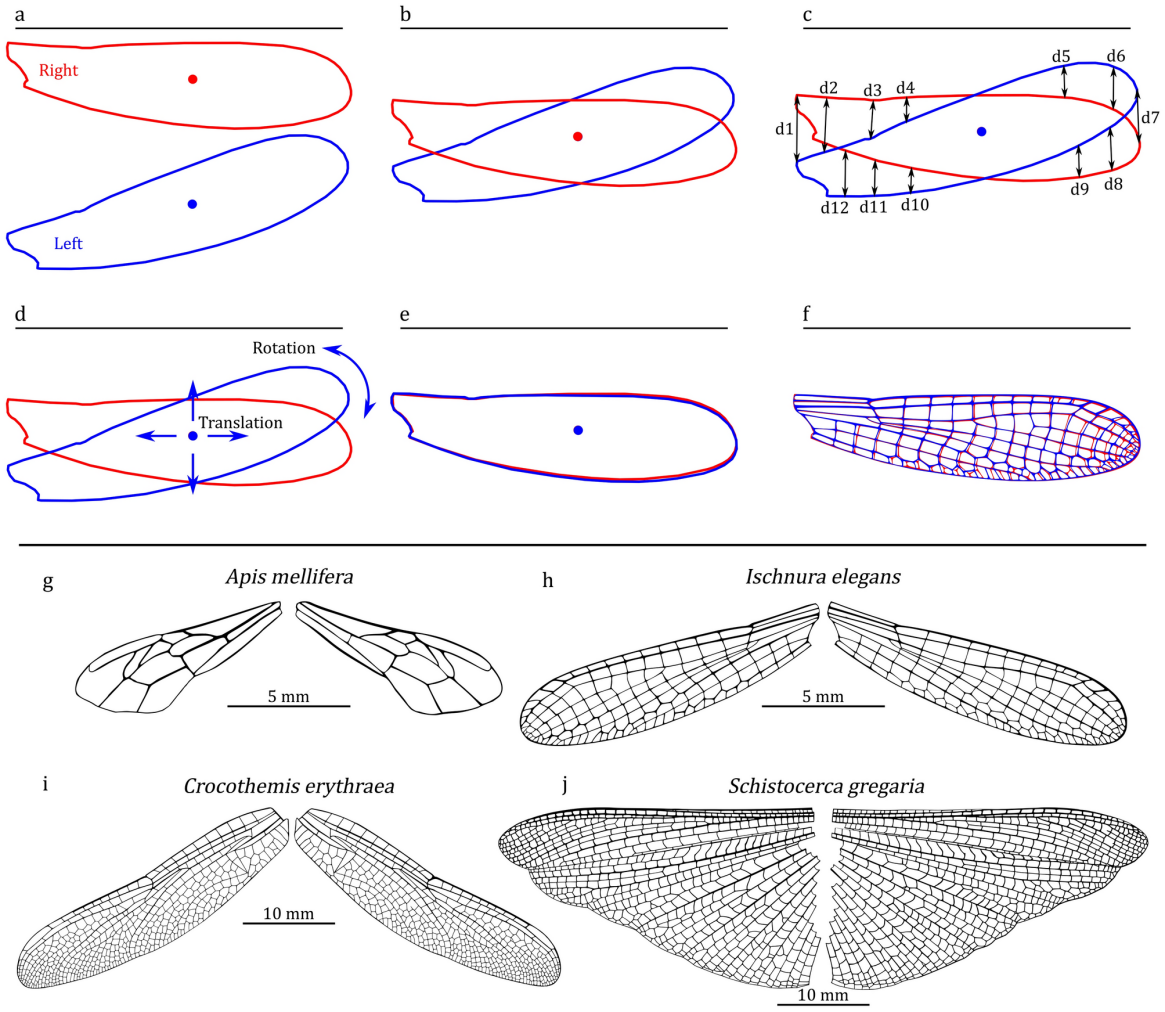
Wing superimposition process and case study selection. (**a**) The outlines of the right and left wings. (**b**) The outlines after aligning them at a common center. (**c**) The measurement of distances between two wing outlines. (**d**) The translation and rotation of one wing for alignment. (**e**) The outlines of two wings after successful alignment. (**f**) Both wings, including their cells, after superimposition. Case study pairs include (**g**) forewings of *Apis mellifera* (Honeybee, Hymenoptera), (**h**) forewings of *Ischnura elegans* (Blue-tailed damselfly, Odonata: Zygoptera), (**i**) forewings of *Crocothemis erythraea* (Scarlet dragonfly, Odonata: Anisoptera), and (**j**) hindwings of *Schistocerca gregaria* (Desert locust, Orthoptera).

Figure 2a illustrates the extracted outlines of the right and left wings. Before initiating the PSO optimization, the centroids of the wings are aligned (Fig. 2b. Subsequently, the method assesses the distances between corresponding points along the wing outlines (Fig. 2c). The PSO’s variables consist of x, y, and theta. ’x’ and ’y’ correspond to translation along the x and y axes, while ’theta’ represents the rotation angle of the wing (Fig. 2d. In each iteration of the optimization process, the particle with the minimal distance is designated as the best particle. After iterative experimentation, it has been determined that the PSO converges to the optimal superimposition after 40 iterations (Fig. 2e. The procedure results in the alignment of the two wings. Subsequently, the method identifies corresponding cells on each wing by calculating the minimum distance between cells, facilitating the quantification of wing asymmetry (Fig. 2f. Code S5, developed for automated superimposition, is detailed in the supplementary information^51^.

### Asymmetry metrics

Once the wings’ boundaries are extracted, the methodology proceeds to quantify the area, length, width, and circularity of the wing cells and the entire wing. The software superimposes wings by employing the PSO technique, thereby establishing correspondence between cells on both wings. The WingAnalogy feature, integral to the software, evaluates wing asymmetry based on several criteria:

1. Normalized root mean square error and regression: The software computes these metrics for the area, length, width, and circularity of cells corresponding to each other. This computation offers insights into the extent of similarity or divergence between the left and right wings.
2. Mean distance measurements: Another criterion for assessing asymmetry involves measuring the distances among the wing outlines, junctions, and centroids of cells.
3. Frequency of cells and junctions: Our methodology quantifies the number of cells and junctions and measures the whole wing’s area, length, width, and perimeter. Utilizing the disparity in these counts and amounts between wings as an asymmetry criterion adds another layer of assessment.

This multifaceted approach gives a wide range of metrics and analyses to evaluate wing asymmetry thoroughly. By encompassing individual cell characteristics and overarching wing features, WingAnalogy provides a comprehensive and detailed perspective on the asymmetry of insect wings. Code S6, developed for computing the asymmetry of two wings, is described in the supplementary information^51^.

### Graphical user interface (GUI)

We have developed a user-friendly graphical interface for those who may not be familiar with coding. This interface is designed using MATLAB App Designer (version 2020b) and contains multiple windows, each serving specific functions. Detailed software information is available in the supplementary information^51^. Moreover, several tutorial videos are available in supplementary files^51^, prepared to show how the software works.

The software encompasses several windows, with Fig. S1 illustrating the main window. In this window, users can create projects from the file and menu (Fig. S2). Each project can include multiple pairs of wings, which can be added using the ’Add New Pair’ button. Clicking this button opens another window, depicted in Fig. S3. Here, users can import wing images and access various Figures related to wing image segmentation and geometric features, similar to what’s shown in Fig. S4. Users can also scale the images (Fig. S3), superimpose them (Fig. S5), and view comparison results through statistics, histograms with distribution fits, and regression graphs (Figs. S6, S7, and S8).

Figure S9 shows a tool embedded in this window that allows users to visualize each wing cell, displaying geometric features such as area, length, width, and circularity. Another tool within this window enables users to divide the wing into a maximum of five sets, each comprising at least three membranes (Figs. S10 and S11).

For each pair, users can generate reports regarding the imported wings and the results of their comparison in various formats, including PDF, CSV, or TXT files. After defining each pair, it is added to the list of pairs in the main window (Fig. S12). After adding several pairs, users can use the ’Result’ menu from the main window to access another window, as illustrated in Fig. S13, to compare all added pairs. In this new window, several comparisons between pairs, such as NRMSE, regression of area, length, width, and circularity, differences in wing areas, and the subtraction of the number of cells and junctions in wing pairs, are available. The EXE file of WingAnalogy can be accessed from the supplementary files^51^.

### Validation, testing, and performance evaluation

WingAnalogy is a multifunctional software featuring numerous windows and functions that seamlessly interact with one another. Several approaches were conducted to assess the software performance, encompassing input and output validation for all functions and evaluating their interactions within the software. WingAnalogy was meticulously designed to be adaptable, accommodating wings from various insect orders, each presenting distinct geometric characteristics and complexities.

One of the fundamental functions within WingAnalogy is insect wing image segmentation, which involves extracting the wing boundary and its constituent cells while measuring their area, length, width, circularity, and the locations of junctions. Due to its pivotal role, this function required thorough validation. Additionally, another critical function of WingAnalogy involves quantifying the asymmetry of wing pairs. In this study, we utilized an image of *Apis mellifera* (honeybee, Hymenoptera) from Fig. 2g to validate both of these functions.

To achieve this, we measured the area, length, and circularity of all cells within the right and left wings of the Honeybee using ImageJ^55^. We then employed our segmentation method within WingAnalogy to measure the same features. Subsequently, we assessed the accuracy of our approach to evaluate its reliability. We utilized the Eq. (2) to compute the accuracy percentage.

2 $$\begin{aligned} Accuracy = 100 \times \frac{|Result_{ImageJ} - Result_{WingAnalogy}|}{Result_{ImageJ}} \end{aligned}$$

Furthermore, we leveraged the results obtained from ImageJ to compute the regression and NRMSE for corresponding cells between the left and right wings, enabling us to quantify the asymmetry of the honeybee wings. We then compared these results to those obtained through WingAnalogy. Moreover, wings from other insect species, including *Ischnura elegans* (Blue-tailed damselfly, Odonata: Zygoptera) (Fig. 2h), *Crocothemis erythraea* (Scarlet dragonfly, Odonata: Anisoptera)(Fig. 2i), and *Schistocerca gregaria* (Desert locust, Orthoptera) (Fig. 2j) are selected to evaluate the performance of the software with more complex wings. The wing images in Fig. 2g–j were acquired using a high-resolution scanner (Epson Perfection V850 Pro). Before their utilization in the software, all images underwent editing.

## Result

The primary focus of this study revolves around the outcomes generated by WingAnalogy software when applied to pairs of insect wing images. In Fig. 2g–j, we have showcased four pairs of wings that serve as our test cases for assessing the software performance in producing results.

**Fig. 3:**
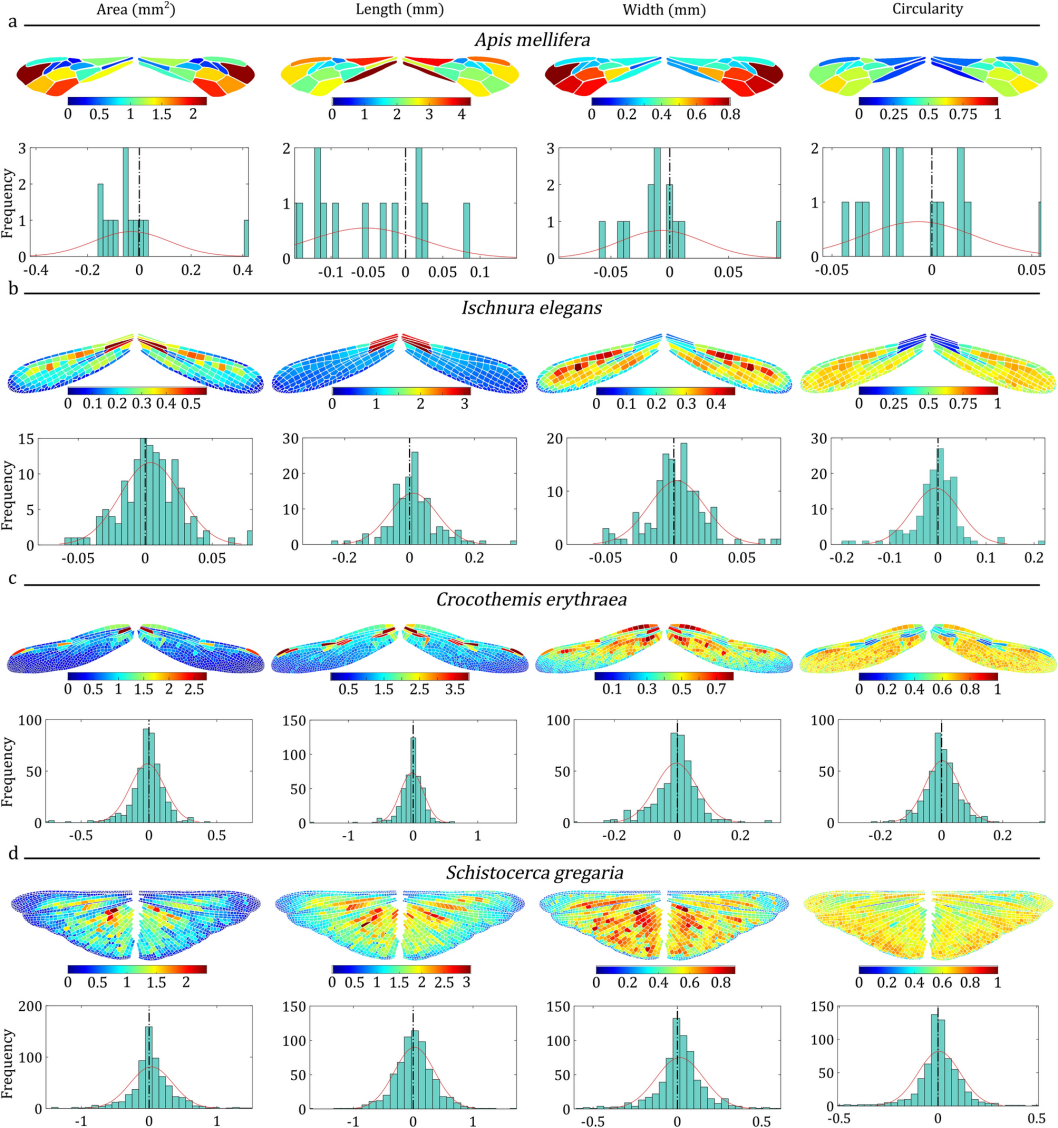
Filled contour plots depicting the distribution of wing cell area, length, width, and circularity, along with corresponding histograms showing a distribution fit for (**a**) *Apis mellifera* (Honeybee, Hymenoptera), (**b**) *Ischnura elegans* (Blue-tailed damselfly, Odonata: Zygoptera), (**c**) *Crocothemis erythraea* (Scarlet dragonfly, Odonata: Anisoptera), and (**d**) *Schistocerca gregaria* (Desert locust, Orthoptera).

**Fig. 4:**
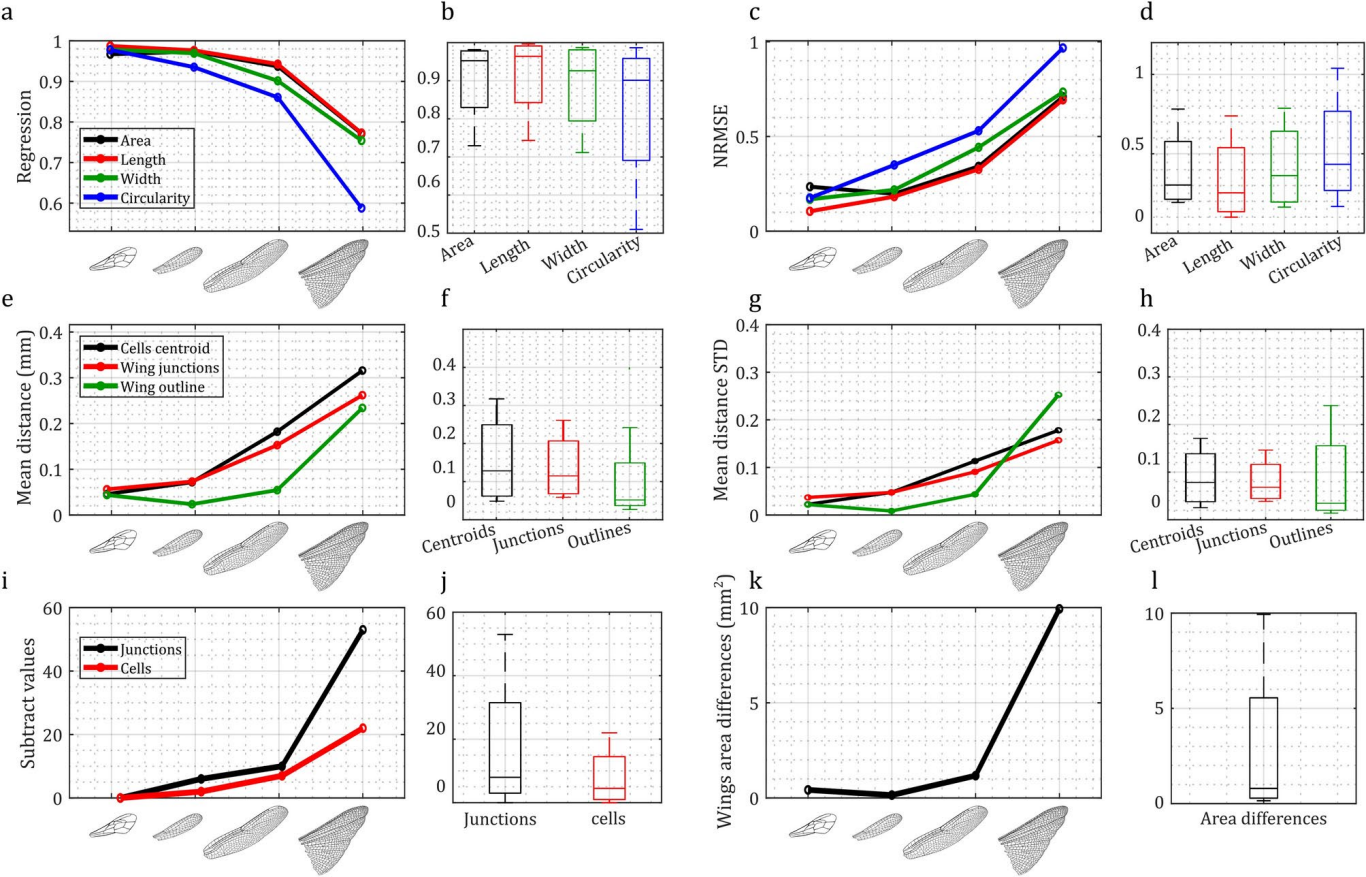
Wing asymmetry comparison for the four case studies of wing pairs. (**a**,**b**) Regressions for area, length, width, and circularity across four wing pairs, and respective boxplots. (**c**,**d**) NRMSE for cell area, length, width, and circularity, and respective boxplots. (**e**,**f**) Mean distances between cell centroids, wing junctions, and wing outlines, and respective boxplots. (**g**,**h**) Standard deviation of mean distances between cell centroids, wing junctions, and wing outlines, and respective boxplots. (**i**,**j**) The subtracted values in the number of cells and junctions and respective boxplots. (**k**,**l**) Wing area and respective boxplot.

**Fig. 5:**
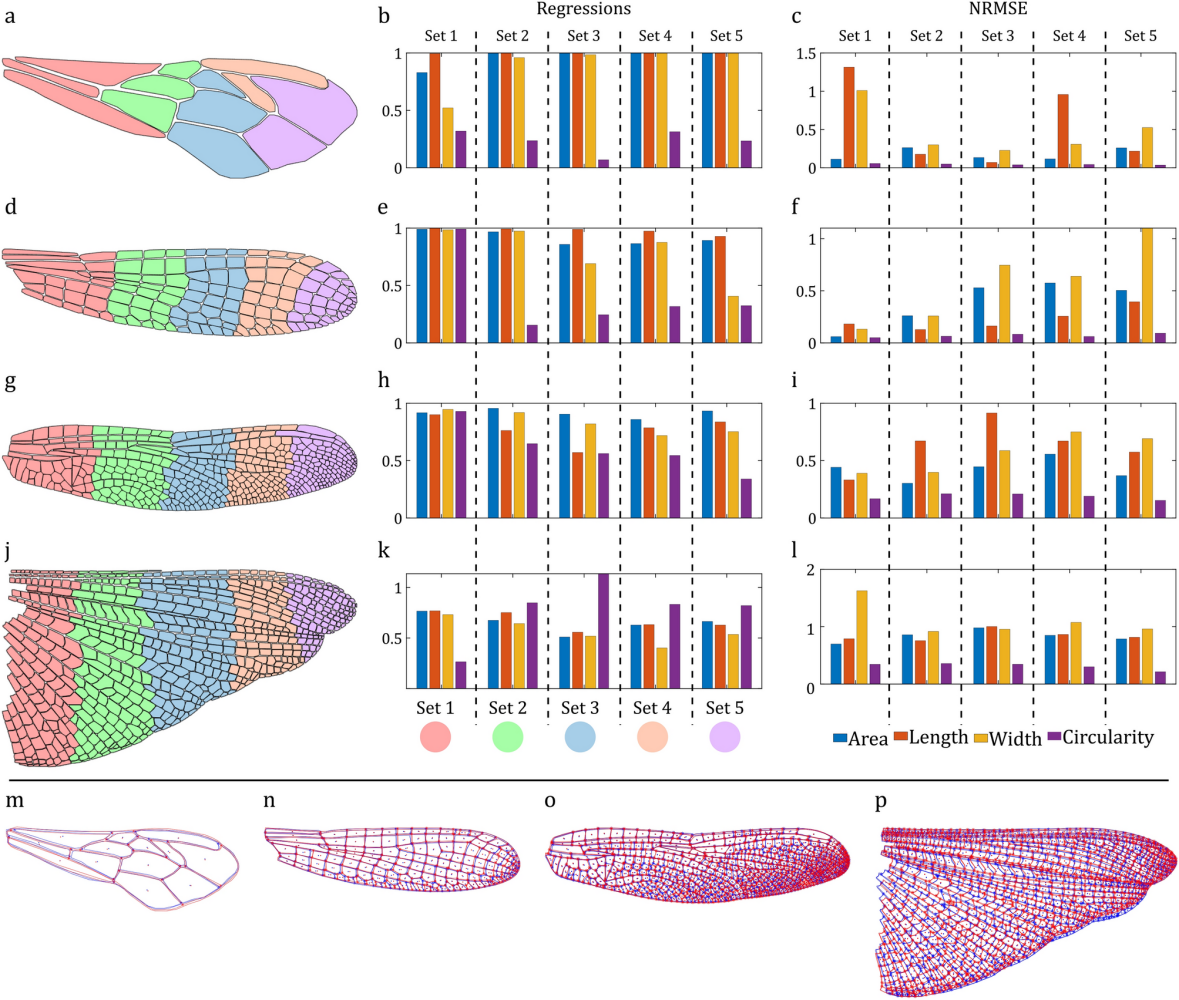
Analysis of wing cell sets and Superimposing Results. (**a**,**d**,**g**,**j**) Display the defined wing cell sets for honeybee, damselfly, dragonfly, and desert locust. (**b**,**e**,**h**,**k**) Depict the regressions of cell area, length, width, and circularity, for the respective insect wing pairs in the case studies. (**c**,**f**,**i**,**l**) Showcase the results of the Normalized Root Mean Square Error (NRMSE) calculations for cell area, length, width, and circularity in the four insect wings studied. (**m**,**n**,**o**,**p**) Illustrate the superimposed wings from each of the four case studies, providing insights into the alignment and comparison of these wing pairs.

Once a new pair is defined and two wing images are added, the software initiates an automated process. It begins by segmenting the wing image, extracting cell boundaries, defining the wing outline, and identifying the locations of junctions. Subsequently, it quantifies parameters, such as the area, length, width, and perimeter of the wing outline and the area, length, width, and circularity of wing cells. Figure 3 shows the contour related to wing cell area distribution, length, width, and circularity generated by WingAnalogy. Beneath each contour, the histogram plot displays the differences in the areas, lengths, widths, and circularities of the left and right wing cells. Additionally, Fig. 4 illustrates the comparison of asymmetry in four different pairs. Next, users can scale the wing images to their actual size and superimpose them for further analysis. Figure 5m-p illustrates the superimposed wings. Additionally, the software allows users to partition the wing into a maximum of five cell sets, as shown in Fig. 5a,d,g, and j. Once these steps are completed, users can generate a comprehensive report for that particular wing pair in three different formats: PDF, TXT, and CSV.

The PDF file contains six chapters, each serving a distinct purpose.

1. Image Specifications (Chapter 1 and 2): These chapters contain information related to the first and second wing image specifications. Details, such as the wing images themselves, cell and junction counts, wing area, perimeter, length, and width, and comprehensive data on cell area, length, width, and circularity, are presented. Contour distributions and histograms illustrating cell characteristics can also be found in these chapters.
2. Comparison Results (Chapter 3 and 4): Chapters 3 and 4 provide insights into the results of comparing wings. Chapter 3 pertains to comparisons with the first image as the reference, while Chapter 4 uses the second image as the reference. These chapters include information on metrics such as NRMSE, regressions, distances between cell centroids, and junctions
3. Results for Wing Sets (Chapters 5 and 6): The final chapters focus on results related to cell sets of the wings. This includes details on the cell sets’ area and regression and NRMSE values based on cell sets’ area, length, width, and circularity.

In the TXT and CSV files, users can access a comprehensive compilation of all quantities and values by comparing wing pairs and image specifications. These files serve as a detailed data repository for further analysis and record-keeping. All PDF, TXT, and CSV files regarding the test cases in this study are available in the supplementary files^51^.

The primary objective of WingAnalogy is to quantify wing asymmetry, which is achieved by extracting geometric features and subsequently computing the results based on predefined asymmetry metrics. This functionality enables the comparison of individual pairs and facilitates the comparison of multiple pairs simultaneously.

Figure 4 illustrates the outcomes of asymmetry measurements generated by WingAnalogy for all four pairs. Figure 4a and c depict each pair regression and NRMSE values, considering parameters, such as area, length, width, and circularity of wing cells. In contrast, box plots in Fig. 4b and d represent the regression and NRMSE values for cell-specific characteristics (area, length, width, and circularity) across all pairs.

Figure 4e provides insights into the mean distance between cell centroids, wing junctions, and wing outlines, serving as an additional metric for assessing wing asymmetry. The boxplot in Fig. 4f visually presents these distances for all pairs. Figure 4g and h mirror the format of Fig. 4e and f, but they pertain to measuring the standard deviation of mean distances.

Furthermore, Fig. 4i offers a visualization of the subtracted values representing the differences in the number of cells and junctions among all pairs. The corresponding boxplot in Fig. 4j displays these values collectively for all pairs. Finally, Fig. 4k and l follow a similar pattern to Fig. 4i and j, focusing on differences in the total wing area among the pairs. The supplementary information^51^ documents the data corresponding to Fig. 4 in Tables S1 and S2.

Additional results are related to the sets of cells depicted within the wings. Figure 5a,d,g and j depict the wings of the honeybee, damselfly, dragonfly, and locust, each divided into five cell sets distinguished by different colors. Next to each wing, the outcomes of set-to-set comparisons are displayed, including regressions and NRMSE values for cells associated with each set (Fig. 5b,c,e,f,h,i,k and l).

**Fig. 6:**
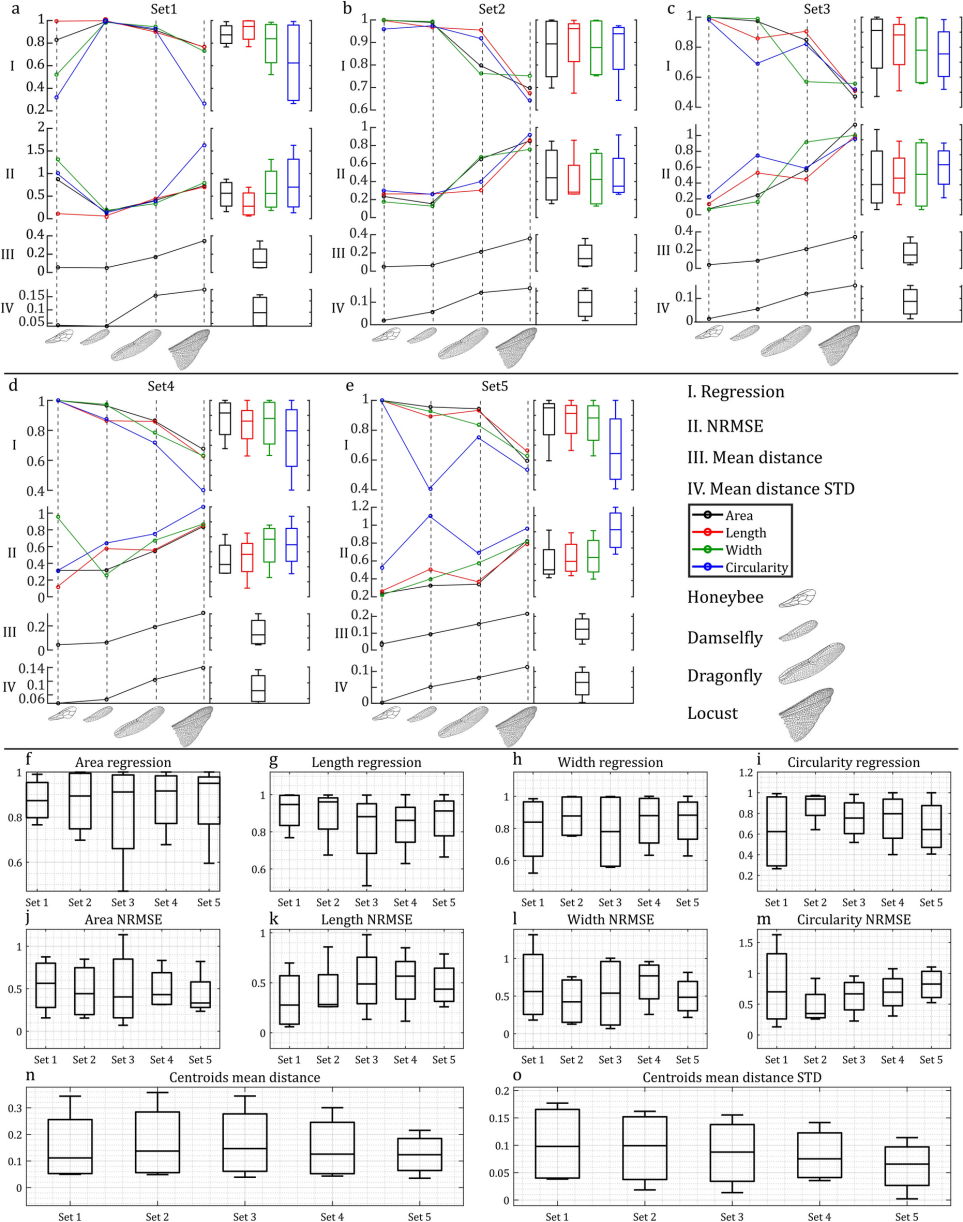
Analysis of defined wing cell sets for the honeybee, damselfly, dragonfly, and desert locust wing pairs. (**a**–**e**) The outcomes of the Regression, Normalized Root Mean Square Error (NRMSE), mean distances of cell centroids, and the standard deviation of mean distances of cell centroids for Sets 1 to 5 across all pairs. (**f**–**i**) Feature boxplots corresponding to the regression of wing cell sets’ area, length, width, and circularity for cell sets 1 to 5. (**j**–**m**) NRMSE values for the area, length, width, and circularity of cells within Sets 1 to 5 for all pairs. (**n**,**o**) The mean distance of wing cell centroids and their standard deviations for wing cell sets 1 to 5 across wing pairs, respectively.

In WingAnalogy, the software capabilities extend to comparing different pairs based on their wing cell sets, as shown in Fig. 6. This Figure offers a comprehensive view of the analytical insights derived from these comparative analyses. Figure 6a–e present detailed results for the first to fifth wing cell sets across all pairs. Within each panel, multiple plots provide distinct insights. Linear plots in these panels correspond to essential metrics, including Regression, NRMSE, Mean Distances of Cell Centroids, and their Standard Deviations. Accompanying each linear plot, a boxplot summarizes the results across all pairs.

Figure 6f–o complement the initial panels with a series of boxplots, each assessing various wing cell sets based on specific metrics. Figure 6f–i showcase boxplots depicting regression values for cell area, length, width, and circularity. Figure 6j–m presents boxplots summarizing NRMSE value for cell area, length, width, and circularity. Figure 5o provides boxplots depicting the mean distances of cell centroids, offering insights into wing cell set comparisons. Figure 6p illustrates boxplots representing standard deviations of mean distances, further enriching set comparisons. Figure 6 provides a comprehensive and structured overview of the results when comparing pairs based on their cell sets within the WingAnalogy software. All the details of Fig. 6 are meticulously documented within Table S3 in the supplementary information^51^.

### Validation

The assessment of WingAnalogy performance was carried out to validate its outcomes and assess its reliability. To achieve this, we employed ImageJ, a renowned tool recognized for its accuracy in image analysis. Our focal point for this validation procedure was the honeybee wing. Consequently, we quantified the area, length, and circularity of all cells within the left and right wings using our developed method and subsequently with ImageJ. The resulting right and left-wing data are stored in Tables S4 and S5 within the supplementary information^51^.

The findings indicate that measurements of area, length, and circularity for the right wing exhibited merely an average difference of 0.056%, 1.59%, and 3.7%, respectively. These results underscore the high accuracy and precision of our methodology. Similarly, in the case of the left wing, average differences in area, length, and circularity were recorded at 0.096%, 1.6%, and 3.8%, respectively. These outcomes further emphasize the reliability and effectiveness of our approach.

Additionally, we conducted assessments of regression and NRMSE between the cell area, length, and circularity of the right and left wings, utilizing results obtained from ImageJ as the benchmark, and subsequently compared these with the outcomes generated by WingAnalogy. Remarkably, the disparities observed in the regression values for area, length, and circularity were merely 0.01%, 0.04%, and 0.1%, respectively. Moreover, the differences in NRMSE for area, length, and circularity were measured at 0.3%, 3.8%, and 3.6%. These findings further underscore that our methodology is as accurate as ImageJ, reaffirming the robustness of our approach.

## Discussion

WingAnalogy is developed to streamline the process of analyzing insect wing geometry and quantifying wing asymmetry. This approach deviates from traditional landmark-based methods, which grapple with inherent limitations. The constraints of landmarking necessitate the precise identification of identical landmarks across all wings, a tough challenge that specific landmarks may be absent due to factors like wing mutations, rendering such wings unsuitable for analysis-despite their potential richness in characters^22,47^. Furthermore, landmark-based approaches encounter difficulties when comparing wings from distinct insect orders, as demonstrated in our case studies. These methods typically focus on a handful of landmarks while overlooking numerous other critical aspects of wing morphology. Additionally, landmark determination often relies on manual input, a time-intensive process that poses challenges for field studies^48–50^.

In contrast, our methodology adopts a multifaceted approach, considering parameters, such as cell area, length, width, circularity, junction locations, cell and junction counts, inter-cell centroid distances, and wing outline attributes (area, length, width, perimeter). This breadth of parameters affords researchers a rich array of analytical options. Moreover, the automated superimposition feature enhances the examination of wing asymmetry. Nevertheless, the process of preparing images for WingAnalogy still involves manual cleaning, which can be time-consuming, especially when dealing with a substantial number of images. We are actively working on enhancing the software to minimize the effort required to prepare images for its use.

While the primary objective of this study is to underscore the eligibility of WingAnalogy, a brief discussion of the results concerning the asymmetry comparison of four case study wing pairs can be illuminating. As our results in Fig. 4 demonstrate, wing asymmetry tends to increase with wing complexity. Notably, the locust hindwings exhibit the highest degree of asymmetry among the case study pairs, whereas honeybee forewings show greater symmetry. Examining the division of wings into five distinct cell sets reveals varying degrees of asymmetry across these cell sets. Of particular interest is the dragonfly wing, which indicates that the base exhibits the highest degree of similarity, with asymmetry increasing along the wing, particularly in terms of circularity-a noteworthy observation signifying that while wing size may not significantly differ, cell shape, as indicated by circularity, exhibits obvious variations along the wing^56^. It is worth mentioning that this study did not employ equal-area divisions within the wings, which may have influenced the results. In subsequent sections, we delve into a comprehensive discussion of WingAnalogy and its versatile applications across different domains.

### Advancements in insect wing analysis

In recent years, insect wing analysis has undergone a transformative evolution, primarily driven by the development of sophisticated software tools^20,25,29,32^. These advancements have revolutionized our approach to studying insect wing morphology and symmetry. Automation and efficiency have become paramount as software automates intricate tasks like image segmentation and cell identification, significantly enhancing the accuracy and speed of analysis. High-resolution imaging technology has enabled researchers to capture intricate wing details, allowing for precise measurements of features and fine-scale asymmetry. Moreover, developing standardized quantitative metrics has expanded our understanding of wing morphology. The ability to perform local comparisons by dividing wings into cell sets has uncovered precise insights, while the capacity for large-scale comparisons has facilitated comprehensive studies across diverse insect populations. Visualization options and detailed reporting further enhance transparency and reproducibility. With interdisciplinary applications and emerging technologies, like machine learning, the future of insect wing analysis holds the potential for uncovering new dimensions of understanding in this field.

### Automation and efficiency

Incorporating automation and efficiency in insect wing analysis represents a significant advancement in our research capabilities. As advanced software tools such as WingAnalogy have come to the forefront, processes that require a significant amount of time and manual effort, such as image segmentation and cell identification, have been streamlined to near perfection. Researchers no longer deal with manually outlining wing structures; automation ensures consistency and precision in every analysis. This automation not only advanced the analytical process but also minimizes human error, resulting in more reliable and reproducible data^20,21,32,57,58^.

### Precision and accuracy

WingAnalogy demonstrates exceptional precision and accuracy in its measurements, as demonstrated by a validation process. To ensure the reliability of its results, a comprehensive validation was undertaken using ImageJ as a benchmark tool. This validation employed the left and right forewings of the honeybee, with individual cell area, length, and circularity measurements conducted independently using both ImageJ and WingAnalogy. Surprisingly, when the results were compared, there was a perfect alignment between the measurements obtained from WingAnalogy and those from ImageJ. This underscores the software’s remarkable ability to replicate measurements with exceptional precision consistently. Moreover, the validation extended to encompass NRMSE and Regression analyses of cell data, where the results obtained from ImageJ were compared with those generated by WingAnalogy, once again revealing an impressive alignment. These findings not only witness to the precision and accuracy of WingAnalogy but also underscore its potential as a reliable tool for researchers in insect wing analysis, ensuring the generation of reproducible data.

While the validation confirms the robustness and accuracy of WingAnalogy, it is essential to note that like all image segmentation methods, ours may introduce small artifacts especially when dealing with low-quality input images. These artifacts are not unique to our method and can occur in manual segmentation or landmarking as well. The quality and resolution of input images play a crucial role in the precision of the analysis. High-resolution images are necessary to capture fine details and ensure accurate segmentation. We recommend a minimum resolution of 300 DPI for scanning insect wings to achieve reliable results. For smaller wings, such as those of *Drosophila*, higher resolutions (e.g. 600 DPI or higher) are advisable. This recommendation ensures that all relevant features are captured accurately, enabling WingAnalogy to perform optimally.

**Fig. 7:**
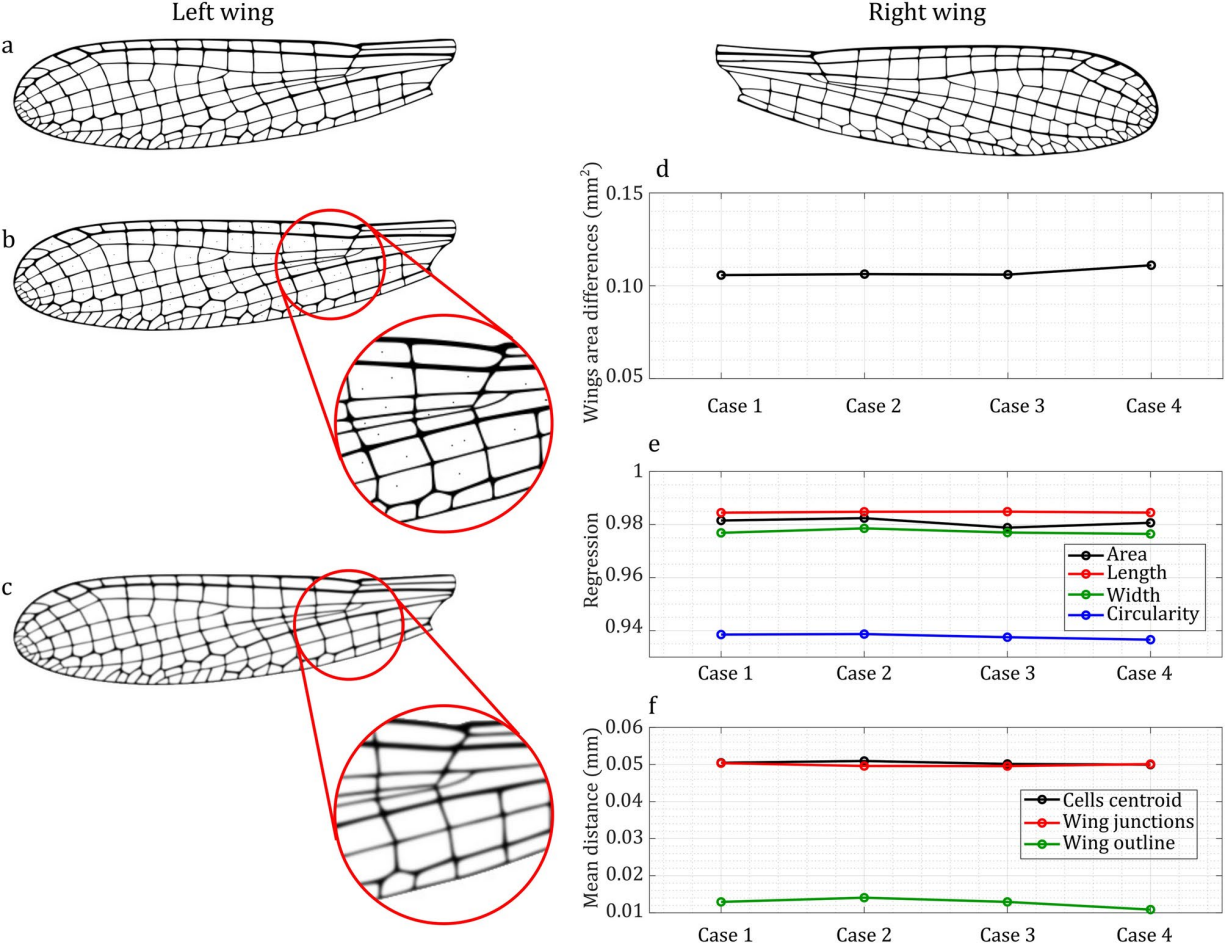
Impact of image quality and artifacts on insect wing asymmetry analysis. (**a**) Case 1: Normal images of the left and right wings of a damselfly at 300 DPI. (**b**) Case 2: Noise added to the left wing image of the damselfly. (**c**) Case 3: The left wing image blurred using Gaussian Blur with a radius of one pixel in Photoshop. Case 4 (not illustrated): Similar to Case 1, but with increased resolution to 400 DPI. (**d**) Comparison of wing area differences. (**e**) Comparison of wing cell area, length, width, and circularity regressions. (**f**) Comparison of wing centroids, junctions, and outline mean distance across the four cases.

However, small artifacts occur in any segmentation process. Figure 7 illustrates that their impact on overall asymmetry analysis is minimal. To demonstrate this, we compared four cases. Case 1 (Fig. 7a) is a normal pair of damselfly wings with a resolution of 300 DPI. Case 2 (Fig. 7b), shows the same pair of wings, but with salt and pepper noise added to the left wing. Case 3 (Fig. 7c), is similar to Case 1, but with the left wing blurred using the Gaussian Blur tool in Photoshop with a radius of one pixel. Case 4, is similar to Case 1, but with a resolution of 400 DPI. Figure 7d shows the results of comparing wing asymmetry based on differences in wing area. Figure 7e presents the results of regressions of cell area, length, width, and circularity. Figure 7f illustrates the mean distances of wing cell centroids, wing junctions, and wing outlines. The results of these plots indicate that small artifacts, such as noise, blurring, or differences in image resolution, do not drastically affect the results of asymmetry analysis.

### Comprehensive data analysis

WingAnalogy offers a comprehensive suite of data analysis tools that collectively provide a comprehensive data analysis of wing asymmetry, providing the diverse needs of researchers in the field. The breadth of data analysis provided by the software encompasses several key solutions for metrics, including regression analysis, NRMSE calculations, centroid distance measurements, and counts of cells and junctions.

- Regression analysis: Regression analysis is a fundamental statistical technique that helps to quantify the relationship between variables. In insect wing analysis, it enables researchers to assess the degree of linear association between corresponding cells in paired wings. By examining the slope of the regression line, users can gain insights into the overall symmetry or asymmetry trends in wing structures. A regression analysis is valuable for identifying systematic deviations from perfect symmetry, providing essential information about the magnitude and direction of asymmetry. The regression analysis aims to compare the morphological metrics of corresponding cells between pairs of wings to quantify asymmetry. A robust approach is used, where each image is treated as a reference in turn, ensuring comprehensive evaluation of all cells. For each pair of wing images, the analysis is performed twice: first using Image 1 as the reference and then using Image 2 as the reference. When Image 1 is the reference, each cell in Image 1 is matched to the closest cell in Image 2 based on centroid distance. This method ensures that, even if Image 2 contains a different number of cells, the cells in Image 1 are adequately matched. In cases where Image 1 has 300 cells and Image 2 has 320 cells, the extra cells in Image 2 are initially ignored. The process is then repeated with Image 2 as the reference to ensure that these additional cells are also considered. This bidirectional matching process helps mitigate the impact of segmentation errors, such as instances where two cells are treated as one or additional cells are detected due to mutations or wing injuries. By performing the regression analysis on the matched sets of cells and using linear regression to compare the morphological metrics (e.g., area, length, circularity) between the corresponding cells, asymmetry patterns can be directly compared and identified. The regression analysis uses the set of points representing the metrics of matched cells rather than the regression equations themselves. This approach allows for a more direct comparison of asymmetry patterns. The system is designed to be resilient to segmentation differences. By using both images as references in turn, the method ensures that all cells are evaluated, thereby reducing the impact of segmentation errors on the overall analysis. This method also accommodates potential biological variations, such as mutations or wing injuries, by ensuring that all detected cells are included in the analysis, either as primary matches or secondary references.
- NRMSE (Normalized Root Mean Square Error) Calculations. NRMSE is a critical metric for evaluating the accuracy of wing superimposition and cell correspondence in WingAnalogy. It quantifies the goodness of fit between corresponding cells in paired wings, measuring the discrepancies between their shapes and sizes. The normalization factor accounts for differences in scale, ensuring that NRMSE considers relative and absolute differences. This metric provides researchers with a quantitative measure of the quality of superimposition and cell matching, aiding in assessing wing asymmetry.
- Centroid distance measurements: WingAnalogy calculates the distances between centroids of corresponding cells and junctions in paired wings. These centroid distance measurements offer insights into the spatial distribution of asymmetry. This information is valuable for understanding the magnitude of asymmetry and its spatial patterns across the wing surface.
- Counts of cells and junctions: Quantifying the number of cells and junctions in paired wings is fundamental to understanding their structural differences. By providing counts of these elements, WingAnalogy offers researchers an essential but crucial metric for characterizing wing morphology. Comparing cell and junction counts between left and right wings can reveal differences that may contribute to overall wing asymmetry.

These metrics provided by WingAnalogy offer a multifaceted view of wing asymmetry, encompassing both quantitative and spatial aspects. Researchers can use these metrics to delve deeply into the structural variations within insect wings, gaining a comprehensive understanding of asymmetry patterns. This complete data analysis capability empowers researchers to conduct thorough investigations into the factors contributing to wing asymmetry, advancing our knowledge in entomology, ecology, and evolutionary biology.

### Wing cell sets analysis

The wing cell sets analysis feature in WingAnalogy is a valuable tool, enabling users to dissect insect wings into distinct sets for localized comparisons. This feature holds great utility in various research scenarios, offering a perspective on wing morphology within and between species. Researchers can utilize this capability to explore intricate variations in different wing cell sets, showing specific adaptations or ecological implications. For instance, when studying wing variation within a single species, this feature facilitates the examination of region-specific adaptations or asymmetry patterns. Additionally, in comparative studies involving multiple species, the wing cell sets analysis allows for identifying species-specific characteristics in particular wing segments, aiding in discerning evolutionary trends and ecological niches.

### Multi pair comparison

The capability of WingAnalogy to compare the results of multiple wing pairs simultaneously is a powerful feature that enhances the scope and depth of research in insect wing analysis. By facilitating the simultaneous analysis of numerous wing pairs within a single project, researchers gain the capacity to draw broader and more robust conclusions. This feature is particularly advantageous when investigating wing asymmetry across various specimens or species. Researchers can leverage the collective data from multiple pairs to identify overarching patterns, trends, or variations. Whether looking at intraspecific variation within a species or interspecific differences between multiple species, this capability allows for a comprehensive exploration of asymmetry dynamics. It provides a broader context for understanding the evolution, ecology, and adaptations of insects, enhancing the depth of insights derived from the wing morphology and asymmetry analysis.

### Report generation

The generation of detailed reports in PDF, CSV, and TXT formats by WingAnalogy holds significance in insect wing analysis. These reports serve as comprehensive and accessible repositories of research findings, streamlining the presentation of results and enhancing the efficiency of data sharing and collaboration among researchers. In PDF format, the reports offer a visual and standardized representation of asymmetry metrics, aiding in clearly and concisely communicating research outcomes. The inclusion of CSV and TXT formats caters to the analytical needs of researchers, allowing for further data processing and statistical analyses. Moreover, these report formats promote transparency and reproducibility by providing a structured and documented account of the analysis process.

### User-friendly interface

WingAnalogy has a user-friendly graphical interface that empowers researchers, regardless of their technical background, to easily access the full potential of the software. Its user-friendly design guides users through each step of the wing analysis process, from project creation and image import to result visualization and report generation. The software offers clear and straightforward options for setting parameters, scaling wing images, and defining wing cell sets of interest, ensuring that users can efficiently tailor their analyses to specific research objectives.

Moreover, the interface provides real-time feedback and visualizations, allowing researchers to preview and validate their selections and configurations, enhancing the accuracy of their analyses. Comprehensive documentation and tutorials further complement the interface, ensuring that users can quickly become proficient in utilizing WingAnalogy for their research needs. The user-friendly interface lowers the barriers for researchers who are not experts in the wing analysis and streamlines the workflow for experienced users, saving time and effort.

## Applications, current limitations and future research

Indeed, discussing the applications and potential future research avenues for WingAnalogy is essential to showcase its versatility and its broader impact on various research domains.

### Applications

1. Taxonomy and species identification: Wing morphology plays a crucial role in species identification and taxonomy^59–61^ WingAnalogy can be a valuable tool for entomologists and taxonomists, enabling them to accurately quantify wing asymmetry and variations within and between species. This can aid in refining species descriptions and understanding evolutionary relationships.
2. Ecology and adaptation: Understanding how wing morphology relates to ecological niches and adaptations is vital. WingAnalogy can help researchers explore how environmental factors influence wing asymmetry and morphology, revealing an understanding of species’ ecological roles and transformations to different habitats^62^.
3. Evolutionary biology: WingAnalogy can contribute significantly to the study of evolutionary biology. Researchers can use it to investigate patterns of wing structure and asymmetry in fossils, allowing for insights into the evolution of insect wings over geological time scales^63,64^.
4. Biomimetics and engineering: In biomimetics and engineering, insights from insect wing analysis can inspire the development of innovative technologies, such as improved aerodynamics, robotics, and drones. WingAnalogy can aid in precisely characterizing natural wing structures for subsequent bio-inspired design^65,66^.

### Current limitations

In the current version of WingAnalogy, the length of cells is defined as the maximum distance between the boundary of each cell. This definition is straightforward and provides a consistent measure of the longest dimension of the cell. However, we acknowledge that this approach might define the length as the diagonal in rectangular cells and that the width, calculated as area/length, can be a poor approximation. To address this, we plan to enhance WingAnalogy in future versions by implementing ellipse fitting for each cell. This will allow us to determine the major and minor axes, providing more accurate measures of length and width. The ellipse-fitting approach will offer a more refined and meaningful representation of cell dimensions, especially for cells with irregular shapes.

Additionally, while circularity is a useful metric, we recognize that it does not provide information about cell anisotropy. Circularity is limited in its ability to describe the elongation or directional properties of cells. To overcome this limitation, we will include metrics that quantify cell anisotropy using an elongation tensor derived from the cell contour. This tensor will provide a norm that quantifies cell anisotropy (aspect ratio), allowing for a more comprehensive analysis of cell shape, independent of wing orientation.

Furthermore, we acknowledge that the current version of WingAnalogy has constraints in image preparation. The software requires high-quality, clean images to function correctly. It does not work well with wings that have transparent veins or dark regions where the differences between membranes and veins are not recognizable. Ensuring that the images are of high resolution and clear distinction between different parts of the wing is essential for accurate analysis.

Moreover, WingAnalogy does not include built-in tools for testing the significance of statistical data. However, it generates comprehensive output data that can be easily exported. For statistical significance testing, users can utilize more dedicated software packages such as SigmaPlot, R, or SPSS. However, this will be a future update to the software.

These enhancements will improve the ability of WingAnalogy to identify sources of left-right asymmetry more effectively, providing researchers with detailed and orientation-independent metrics for size and shape. Future versions will also aim to mitigate current image preparation constraints by incorporating more advanced image processing techniques to handle a wider variety of wing images.

### Future research avenues

1. Morphological evolution: Future research could explore how particular wing morphology has evolved within and across insect taxa. This could involve analyzing fossil wings and using WingAnalogy to compare them with extant species, providing insights into long-term morphological trends.
2. Eco-morphology: Exploring the connections between wing structure, ecological habitats, and behavior can deepen our understanding of insect ecology. WingAnalogy can be employed to examine how wing characteristics are linked to specific ecological niches.
3. Climate change impacts: With climate change affecting insect populations, researchers can use WingAnalogy to assess how changing environmental conditions influence wing morphology. This can provide early warnings of ecological disruptions.
4. Intraspecific variation: Studying intraspecific variation in wing morphology using WingAnalogy can reveal valuable information about local adaptations and population dynamics. This could be particularly relevant in understanding how insects respond to environmental gradients.
5. Biodiversity conservation: The software’s applicability extends to biodiversity conservation efforts, where researchers can leverage it to examine wing asymmetry in threatened or endangered species. This utilization assists in enhancing conservation strategies and monitoring efforts. More specifically, asymmetry can serve as an informative indicator of population health.
6. Machine learning integration: Future developments might include integrating machine learning techniques for automated species identification based on wing morphology, further streamlining taxonomy and fieldwork.

In conclusion, WingAnalogy presents a versatile tool for insect wing analysis with applications across various research domains. Its potential for enhancing our understanding of insect morphology, ecology, and evolution is substantial, and ongoing and future entomological research using this software can continue to expand the boundaries of evolutionary and ecological knowledge.

## Supplementary Information

Supplementary Information.

## Data Availability

We have included Supplementary Information and Supplementary Files to provide a comprehensive and detailed account of our study. The Supplementary Information, available in PDF format, encompasses additional figures, detailed descriptions of principal codes, and the Graphical User Interface of WingAnalogy. All the materials in the Supplementary Information, including codes, Figures, Sample Images, Videos, and Installable Software files, are available via the Zenodo repository. The repository can be accessed using the following link: https://zenodo.org/doi/10.5281/zenodo.7156257.
