# Supplementary material for: WingAnalogy: a computer vision-based tool for automated insect wing asymmetry and morphometry analysis
Source: Sci Rep. 2024 Sep 27;14:22155. doi: 10.1038/s41598-024-73411-x (PMC11437043; doi:10.1038/s41598-024-73411-x)
Supplement: Supplementary file 1 — Supplementary Information. [file 41598_2024_73411_MOESM1_ESM.pdf]

# Supplementary information for
# "WingAnalogy: A Tool for Automated Insect Wing Asymmetry and Morphometry Analysis"

Shahab Eshghi*[1], Hamed Rajabi[2,3], Natalia Matushkina[4], Lisa Claußen[1], Johannes Poser[1], Thies H. Büscher[1], and Stanislav N. Gorb[1]

[1]*Functional Morphology and Biomechanics, Institute of Zoology, Kiel University, Kiel, 24118, Germany*

[2]*Division of Mechanical Engineering and Design, School of Engineering, London South Bank 6 University, London, UK 7*

[3]*Mechanical Intelligence Research Group, South Bank Applied BioEngineering Research 8 (SABER), School of Engineering, London South Bank University, London, UK*

[4]*Institute of Biology and Medicine, Taras Shevchenko National University of Kyiv, Kyiv, Ukraine*

These supplementary materials encompass Figures, tables, and links that direct you to the Zenodo repository, which hosts the installable files and additional supplementary resources.

## Contents

**1 Graphical User Interface** **2**

**2 Validation** **9**

**3 Tables** **10**

**4 Supplementary Codes** **13**

4.1 Code S1: Get Image . . . . . 13
4.2 Code S2: Region Growing . . . . . 14
4.3 Code S3: Wing Cell Segmentation . . . . . 16
4.4 Code S4: Junction Detection/Skeletonization . . . . . 18
4.5 Code S5: Automated Superimposition Using Particle Swarm Optimization (PSO) . . . . . 20
4.6 Code S6: Computing Asymmetry . . . . . 23

**5 Supplementary Files (Zenodo Repository)** **27**

5.1 Supplementary Codes . . . . . 27
5.2 Supplementary Figures . . . . . 27
5.3 Supplementary Sample Images . . . . . 28
5.4 Supplementary Videos . . . . . 28
5.5 Other Supplementary Files . . . . . 28

*Corresponding author: eshghi.shahab@gmail.com

# 1 Graphical User Interface

WingAnalogy is a software designed for measuring the asymmetry of insect wings. Figure S1 shows the main window of WingAnalogy. Defining a new project is the first step in using the software. To initiate a new project, users should begin navigating to File > New Project, thereby defining the project properties using the *New Project* window, as demonstrated in Figure S2.

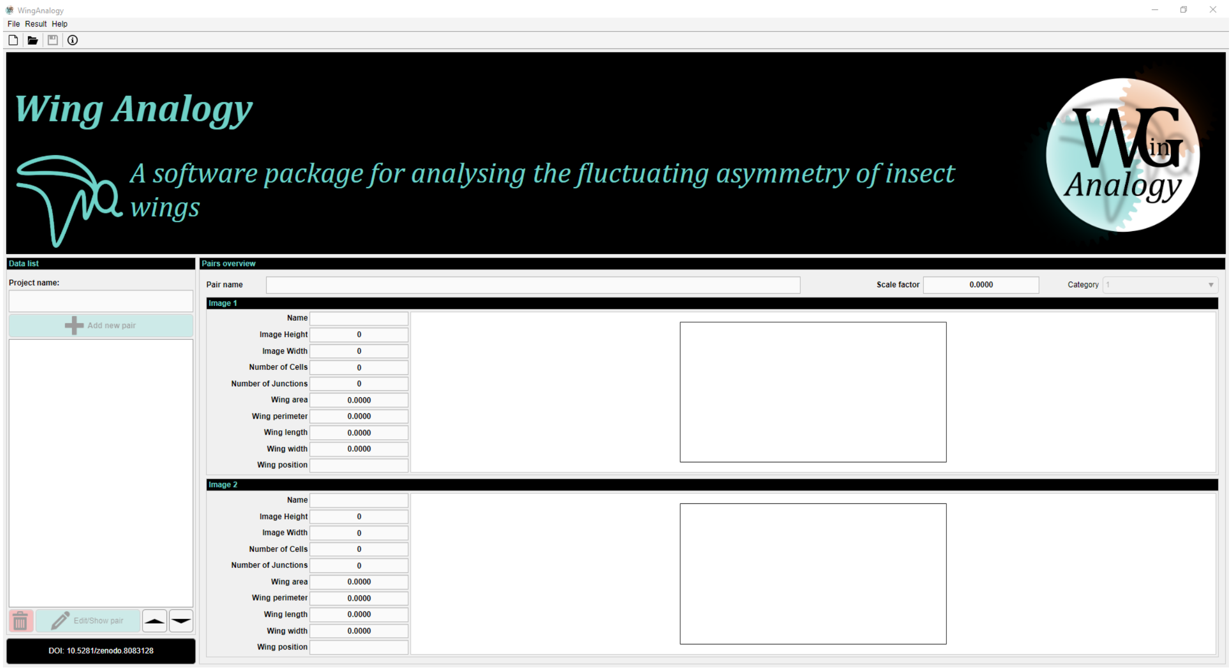


Figure S1: The main window of WingAnalogy displays a list on the left side containing the names of imported pairs. On the right side, the user can view the selected pair name, the names, and sizes of the imported images, the number of cells and junctions in each image, as well as the perimeter, length, width, and area of the wing. Additionally, the scale factor related to the pair is provided.

In the *New Project* window, as illustrated in Figure S2, the user should select a directory on their computer where all files related to the project will be stored. Additionally, for each project, the user needs to assign a name to the project, specify the project manager, and provide their email address. Furthermore, there is a text box where the user can input any important notes about the project.

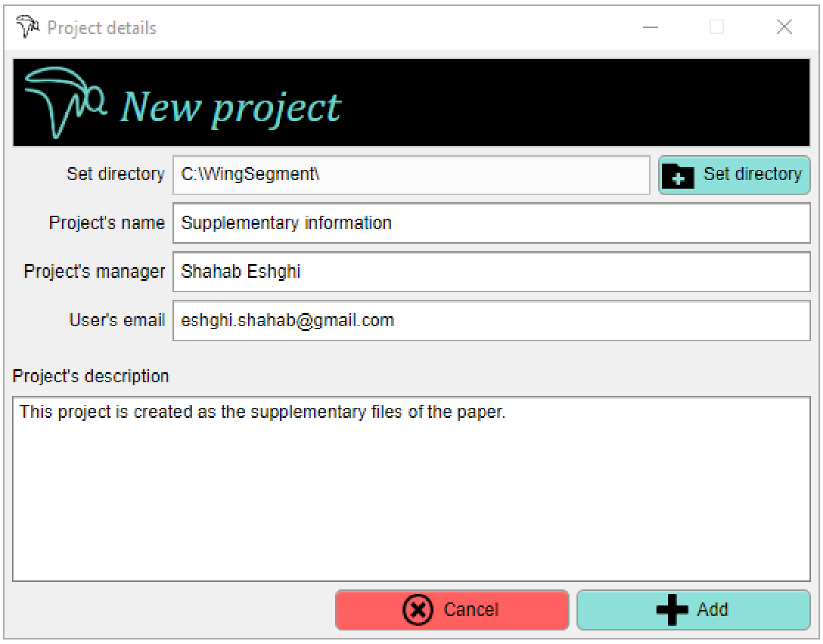


Figure S2: *New project* window. In this window, the user can define the project and provide all relevant related information.

After defining the new project, the user can initiate the process of measuring wing asymmetry by clicking the

*Add New Pair* button. Clicking this button opens a new window, as illustrated in Figure S3. This window is named *Add/Edit Pair*. In this window, the user must import two insect wing images to be compared. For this purpose, an *Add Image* button is provided. After both images are imported, from the options panel on the right side in Figure S3 under the *Scaling* label, the user can scale the imported wing to its actual size. To do this, the user should adjust the line to match the corresponding length of the wing and enter the corresponding real length of the wing in the dedicated text area for this purpose.

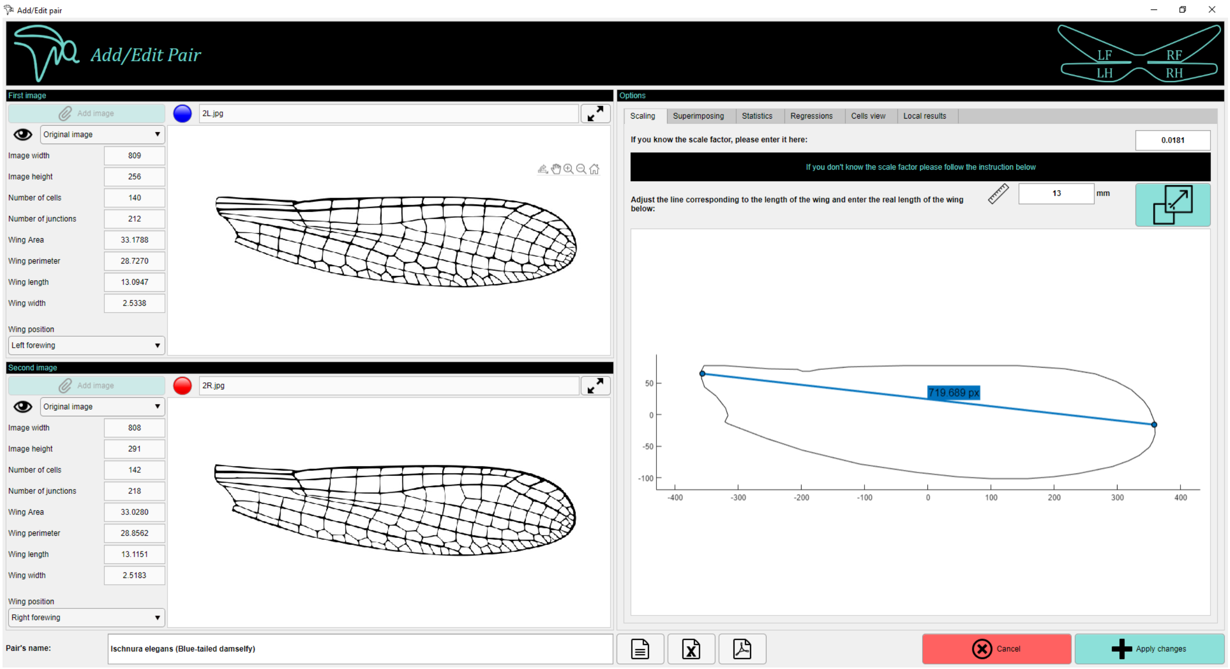


Figure S3: *Add/Edit Pair* window of WingAnalogy software

When an image is imported, the built-in algorithms segment the insect wing image and measure geometric features related to the wing and its cells. As a result, several Figures are accessible for each wing. By using the drop-down under the *Add Image* button, you can switch between different Figures such as the Binary image, the Skeletonized image, the outline of the wing, extracted cells and junctions, the histogram and distribution contour related to the cell area, length, width, and circularity which are illustrated for this example in Figure S4. Additionally, as shown in Figure S3 on the right side of the image name, a button allows you to maximize the Figure for a detailed view. Information regarding the image size, number of cells, junctions, wing area, perimeter, length, and width is displayed on the left side of the display panel for each wing. Furthermore, for each wing, the wing position can be defined.

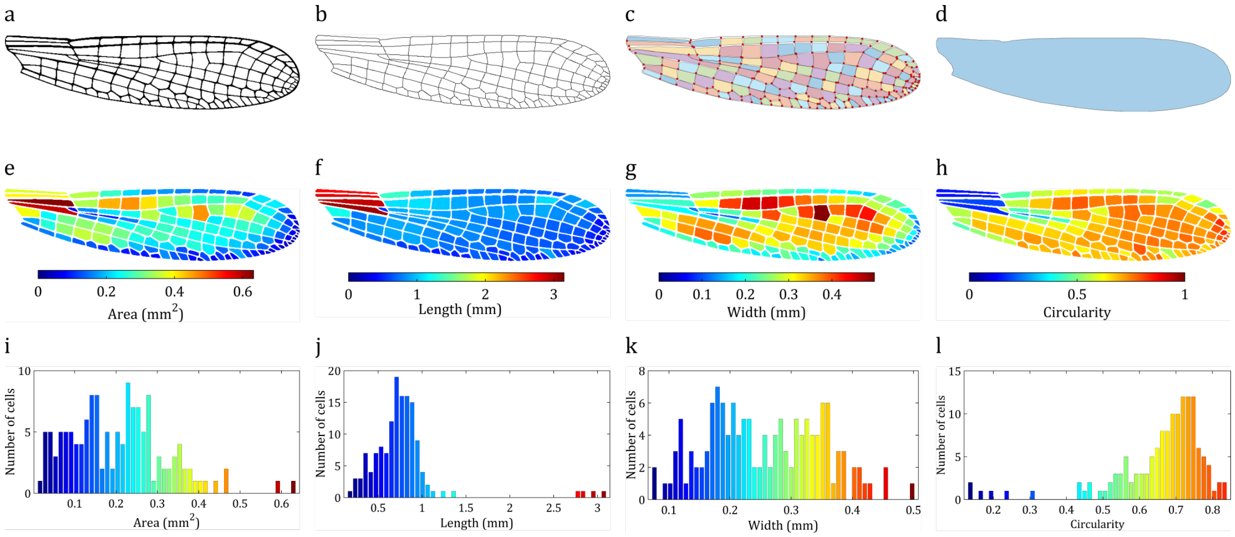


Figure S4: Generated Figures by WingAnalogy for a desired wing: a) Binary image. b) Skeletonized image. c) Extracted cells and junctions. d) Extracted wing outline. e, f, g, h) Distribution of cell area, length, width, and circularity, respectively. i, j, k, l) Histogram of cell area, length, width, and circularity, respectively.

The next crucial step is superimposing the wings. In the *Add/Edit Pair* window, this tool can be located in the options panel under the *Superimposing* label, as depicted in Figure S5. The display panel in this section presents both

wings in red and blue colors, with the blue image being the reference. Users can use the arrows to reposition the red wing until it is properly superimposed. Additionally, a button labeled *Auto Superimposing* is available in this panel, which can automatically align the wings. We recommend using the auto-superimposing feature. If it does not work properly, you can try aligning the wings manually.

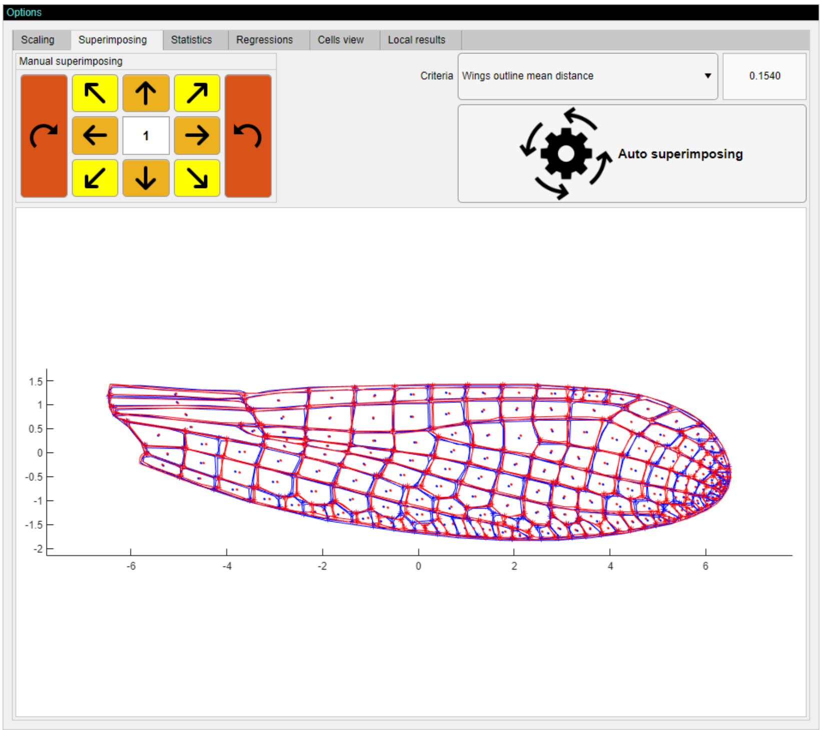


Figure S5: Superimposing tool within the *Add/Edit Pair* window. Arrow buttons are provided for manual superimposing. Additionally, there is an automated superimposing button for streamlining the process. The display panel presents both wings in different colors: blue and red.

After superimposing, as illustrated in Figure S6, you can observe the results related to the wing comparison through the *Statistics* labels in the *Options* panel. The *Statistics* label includes the NRMSE and regressions concerning the wing cell Area, Length, Width, and Circularity. It also provides the mean distance and standard deviations of mean distances between wing outlines, junctions, and cell centroids. Additionally, the subtracted values of the junctions and cells are presented here. In the *Statistics* label, there are three columns of results labeled as *Mean Value*, *Image 1*, and *Image 2*. It is important to note that WingAnalogy considers the first image as the reference image in one scenario and the second image in another scenario. Consequently, when presenting the results, both sets of results are presented, along with the mean value calculated from them.

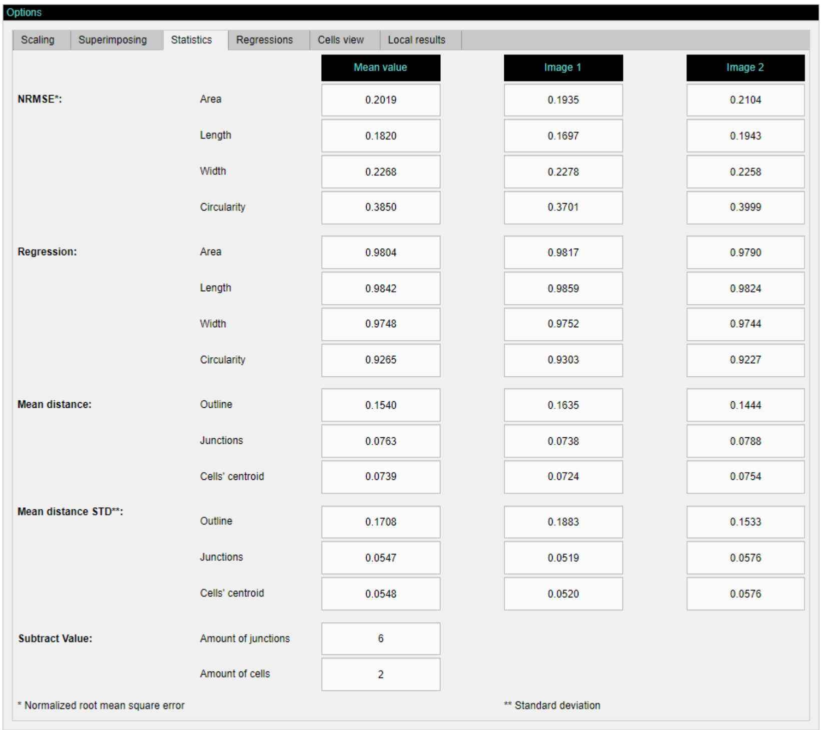

Options

Scaling | Superimposing | Statistics | Regressions | Cells view | Local results

| | | Mean value | Image 1 | Image 2 |
|---|---|---|---|---|
| NRMSE*: | Area | 0.2019 | 0.1935 | 0.2104 |
| | Length | 0.1820 | 0.1697 | 0.1943 |
| | Width | 0.2268 | 0.2278 | 0.2258 |
| | Circularity | 0.3850 | 0.3701 | 0.3999 |
| Regression: | Area | 0.9804 | 0.9817 | 0.9790 |
| | Length | 0.9842 | 0.9859 | 0.9824 |
| | Width | 0.9748 | 0.9752 | 0.9744 |
| | Circularity | 0.9265 | 0.9303 | 0.9227 |
| Mean distance: | Outline | 0.1540 | 0.1635 | 0.1444 |
| | Junctions | 0.0763 | 0.0738 | 0.0788 |
| | Cells' centroid | 0.0739 | 0.0724 | 0.0754 |
| Mean distance STD**: | Outline | 0.1708 | 0.1883 | 0.1533 |
| | Junctions | 0.0547 | 0.0519 | 0.0576 |
| | Cells' centroid | 0.0548 | 0.0520 | 0.0576 |
| Subtract Value: | Amount of junctions | 6 | | |
| | Amount of cells | 2 | | |

* Normalized root mean square error

** Standard deviation

Figure S6: Statistics related to comparing wing images can be found in this label. It includes several statistics related to the NRMSE and regression of cell area, length, width, and circularity, as well as the mean distance and standard deviation between wing outlines, junctions, and cell centroids.

The next label, as shown in Figure S7, is the *Distribution Histogram*, which displays the histogram depicting the differences in the corresponding wing cell area, length, width, and circularity. Additionally, the Figures related to regressions are presented under the *Regressions* label in the Options panel, as illustrated in Figure S8.

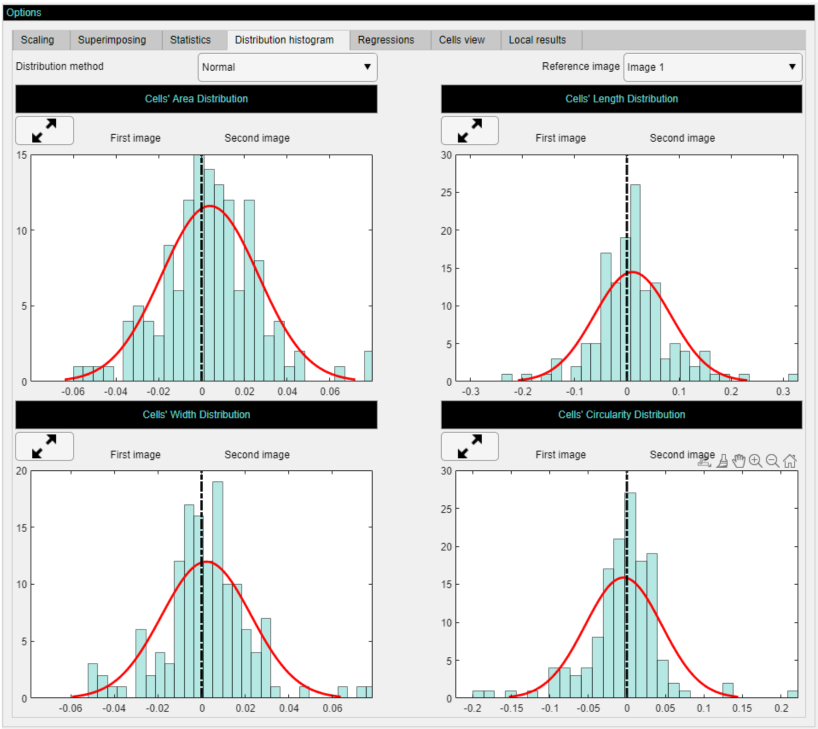


Figure S7: Histogram of cell area, length, width, and circularity with a distribution fit.

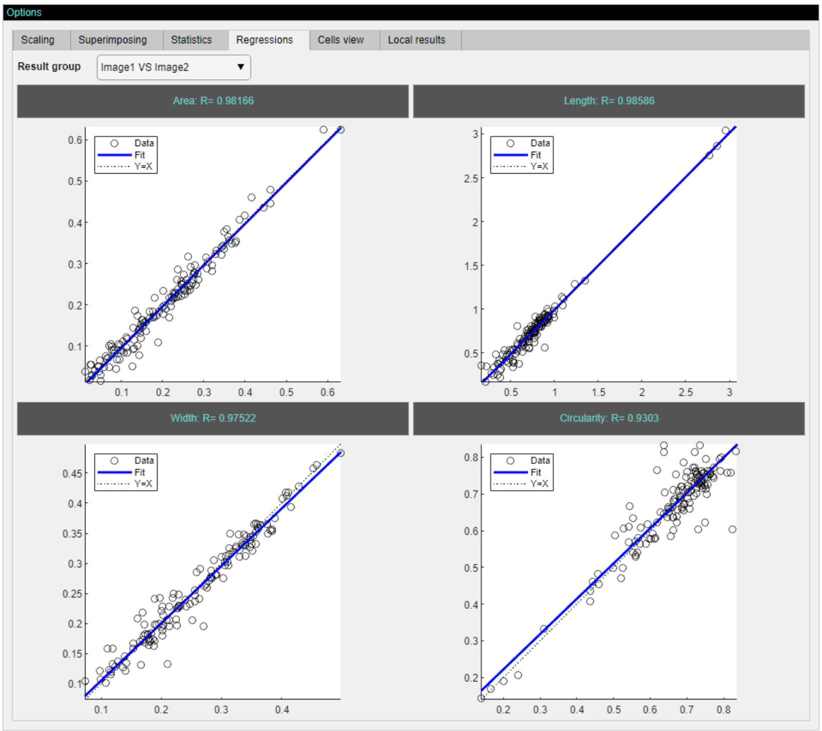


Figure S8: Graphs related to the regression of wing cell area, length, width, and circularity.

Another tool, which is illustrated in Figure S8 and embedded in the Options panel, is the *Cell View*. As mentioned before, when the user imports each image, built-in algorithms segment the wing image and measure the area, length, width, and circularity of wing cells. Consequently, in this panel, the user can view these measurements for each cell from either the first or second image separately.

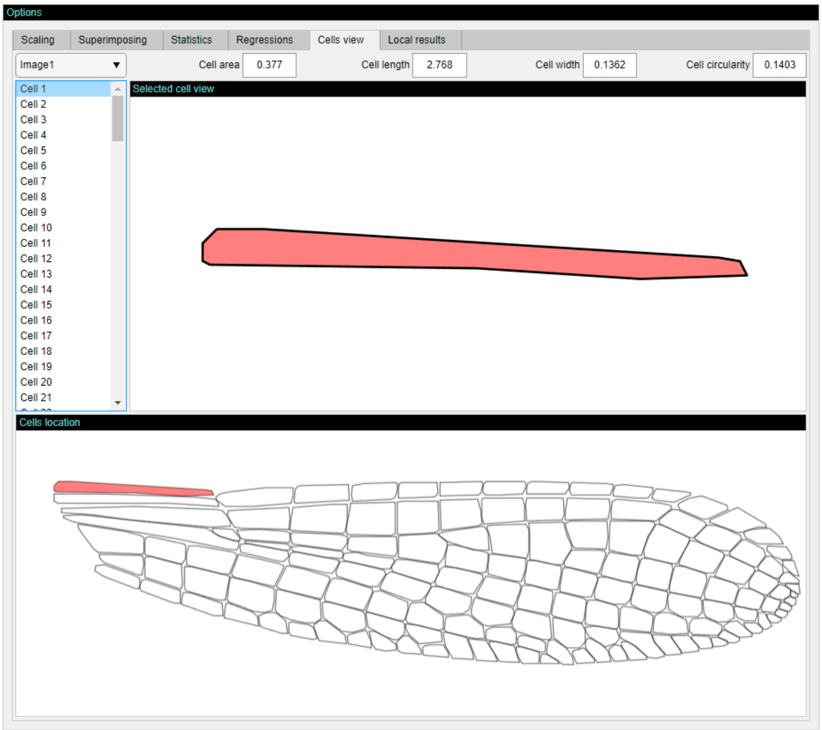


Figure S9: Cell View Tool: This tool allows the user to view the area, length, width, and circularity of individual cells. It includes a dropdown menu for changing the displayed image.

WingAnalogy is not only developed for comparing entire wings, but it also allows for localized comparisons. This means that we have included a tool in which the user can divide the wing into a maximum of five different regions and obtain results for each region separately. This tool is accessible under the *Local Results* label in the Options panel. As mentioned earlier, the software can consider either the first image or the second image as the reference. Then the user can freely choose one of the images to divide the wing into several regions. Clicking the *Edit/View Sets* button opens a new window with several embedded tools, enabling the user to specify arbitrary regions within the wing and

obtain results for each.

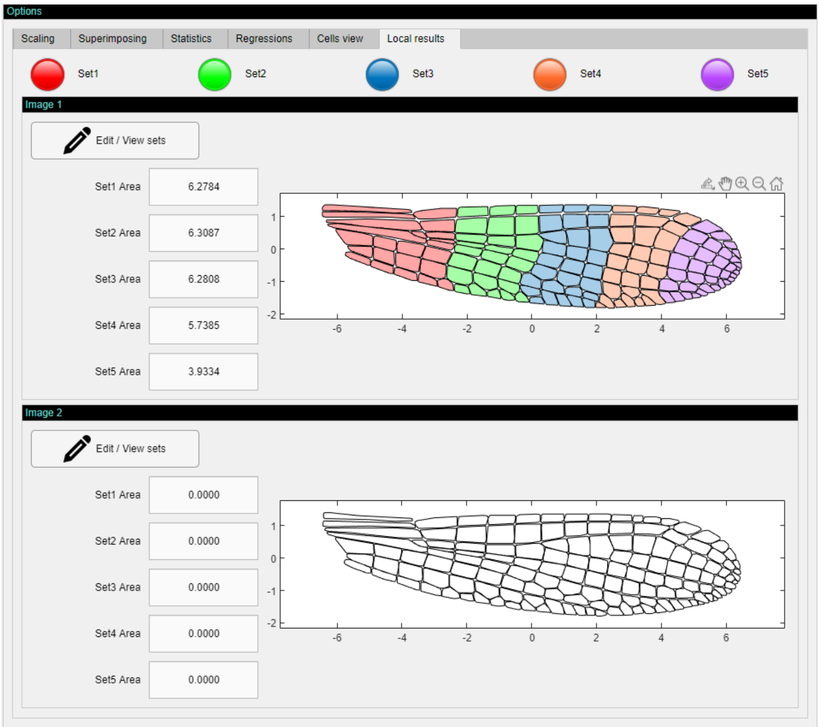

Figure S10: Under the *Local Results* label, the user can access another window, as illustrated in Figure S11, where they can define specific regions within the wing and perform localized wing comparisons.

Figure S11 displays the Local Result window. In this window, the user can access several tools for dividing the wing into a maximum of five sets. There are blue and red buttons for each set under the *Define Set* panel. The blue button activates the mouse cursor, allowing the user to outline a region on the wing by drawing a polygon around it. The centroid of each cell inside the selected region is considered part of that set. Once a set is defined, the area of that set appears in the dedicated text box in front of it. Additionally, as soon as one set is defined, the color of that region changes to correspond with the set color, which is defined as red, green, blue, orange, and purple, respectively, for sets one to five. Furthermore, the results are displayed immediately after defining a set under the *Sets Overall Result* and *Statistics* panel.

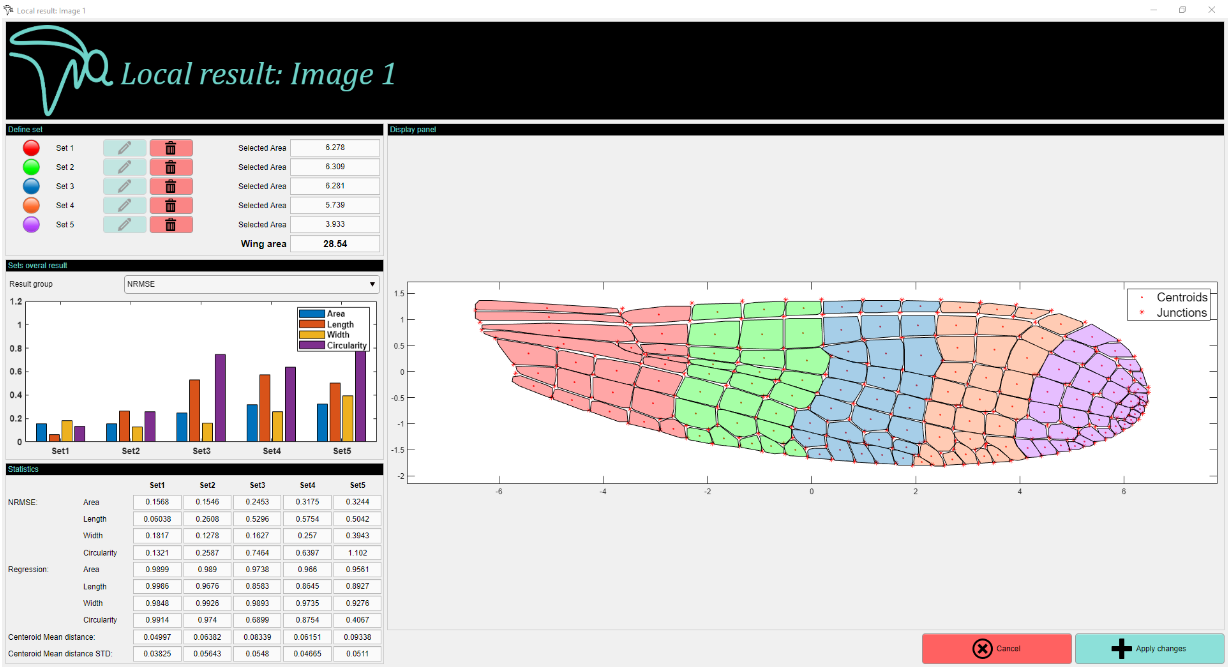

Figure S11: The Local Result window provides the user with access to tools for defining up to five sets and obtaining results from different regions.

After completing all tasks related to wing pairs, the user can utilize three embedded buttons under the *Options*

panel in the *Add/Edit Pair* window, as illustrated in Figure S3, to generate a report in three different formats: TXT, PDF, or CSV files. By clicking *Apply Changes* in Figure S3, the pair is added to the list of pairs on the left side of the main window, as shown in Figure S1. Figure S12 displays the main window after four pairs have been added to the software.

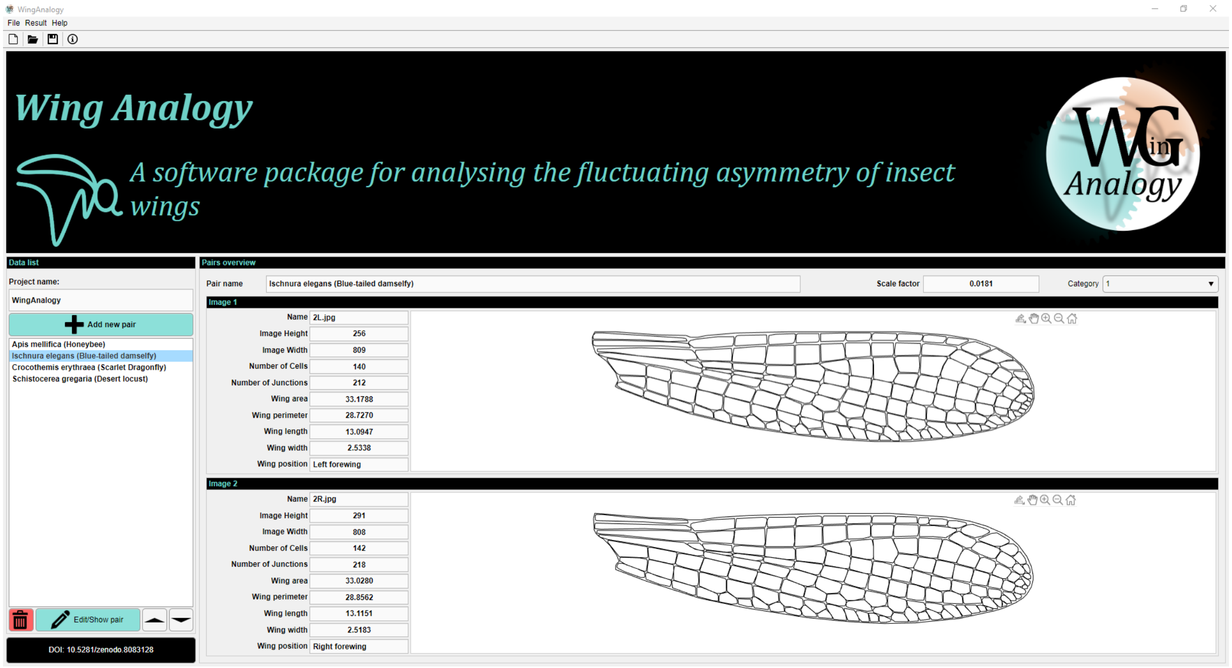


Figure S12: The main window of WingAnalogy after defining four pairs.

Clicking on the name of a pair, as shown in Figure S11, displays the images of that pair. The user can click the *Edit/Show Pair* button to return to the window shown in Figure S3 and adjust information related to the pair.

In the main window, the user can access the *All Pairs Result* window via the *Result* menu, as illustrated in Figure S13. This window compares the results of regression, NRMSE, mean distances, standard deviation of mean distances, subtract values, and wing area differences for all pairs through simple plots and box plots. All defined pairs are displayed on the left side. The numbers on the x-axis of the simple plots correspond to the pair numbers listed. The menus at the top of this window offer the user several options, including exporting Figures and their data as CSV or PDF files.

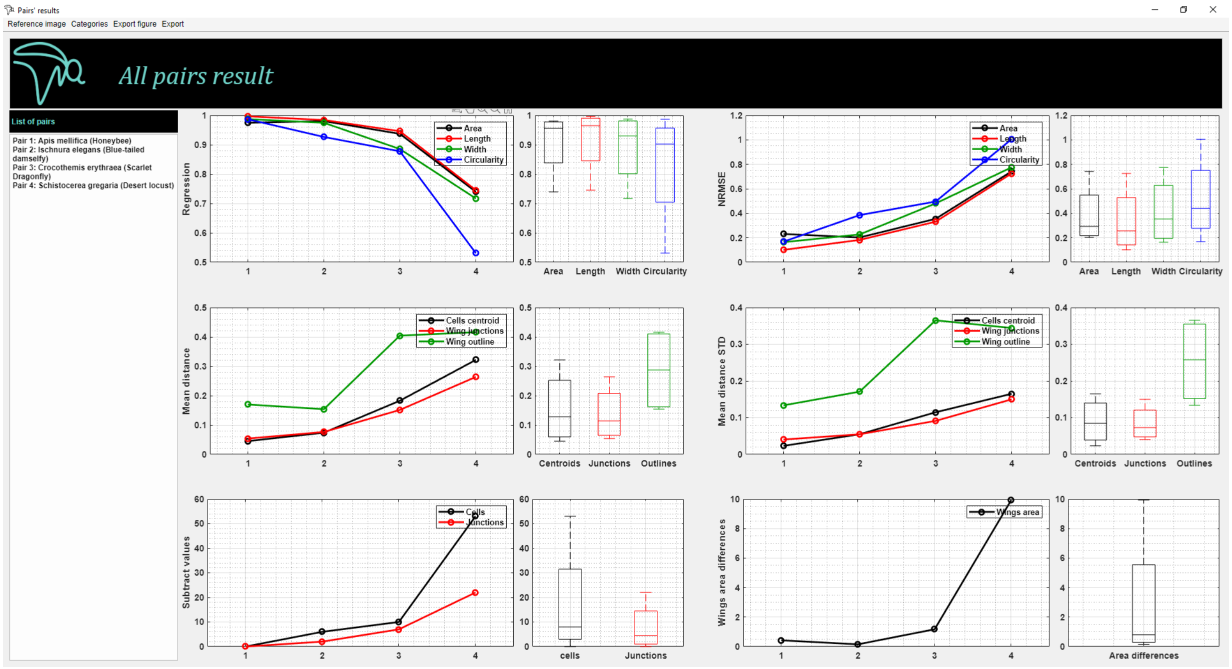


Figure S13: All pair result Window.

## 2 Validation

Figure S14 displays the left and right wings of a honeybee, with labeled cells for reference. Tables S1 and S2 present the results of area, length, and circularity measurements for the right and left wings in Figure S14. These tables include measurements obtained using both WingAnalogy and ImageJ. The last three columns in both tables represent the percentage differences between the measurements from both methods. The overall findings indicate a close alignment between the two methods, providing strong evidence of the reliability of our measurement approach.

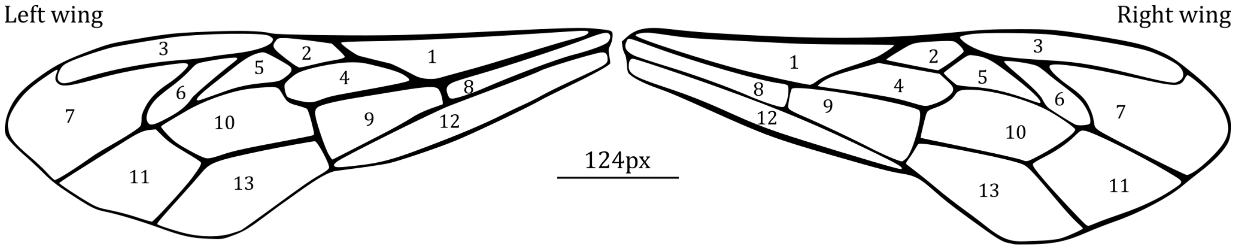


Figure S14: Visualization of the left and right wings of a honeybee with labeled cells. The labeling is intended to correspond to geometric information presented in table S4 and S5, providing insights into the characteristics of wing cells.

# 3 Tables

The tables provided in this section present the documented values corresponding to the results and Figures presented in the main manuscript. Specifically, Table S1 contains regression and NRMSE values for area, length, width, and circularity of wing cells in *Apis mellifera* (honeybee, Hymenoptera; forewings), *Ischnura elegans* (blue-tailed damselfly, Odonata: Zygoptera; forewings), *Crocothemis erythraea*(Scarlet dragonfly, Odonata: Anisoptera; forewings), and *Schistocerca gregaria* (desert locust, Orthoptera; hindwings), along with mean distances, standard deviations of mean distances, subtracted values of cell and junction numbers, and differences in wing areas. These results serve as the basis for Figure 3 in the main text. Table S2 encompasses measurements made by WingAnalogy, including wing area, length, width, perimeter, cell and junction counts for the left and right wings of honeybee, damselfly, dragonfly, and locust. Table S3 presents asymmetry values for defined sets of wings, which are subsequently visualized in Figures 4 and 5 in the main text. Tables S4, S5, and S6 contain validation results for WingAnalogy, focusing on wing image segmentation and asymmetry measurement. These validation tests were conducted using honeybee wing images.

Table S1: The values that correspond to those displayed in Figure 3.

| Asymmetry metrics | *Apis mellifera* | *Ischnura elegans* | *Crocothemis erythraea* | *Schistocerca gregaria* |
|---|---|---|---|---|
| Regression(Area) | 0.9748 | 0.9804 | 0.9372 | 0.7394 |
| Regression(Length) | 0.9967 | 0.9841 | 0.9459 | 0.7453 |
| Regression(Width) | 0.9871 | 0.9748 | 0.8854 | 0.7169 |
| Regression(Circularity) | 0.9867 | 0.9265 | 0.8777 | 0.5309 |
| NRMSE(Area) | 0.2307 | 0.2019 | 0.3545 | 0.7419 |
| NRMSE(Length) | 0.1009 | 0.1820 | 0.3310 | 0.7251 |
| NRMSE(Width) | 0.1637 | 0.2268 | 0.4803 | 0.7752 |
| NRMSE(Circularity) | 0.1684 | 0.3850 | 0.4955 | 1.0051 |
| MD(Cells centroid) | 0.0452 | 0.0739 | 0.1827 | 0.3218 |
| MD(Junctions) | 0.0539 | 0.0763 | 0.1514 | 0.2638 |
| MD(Wing outlines) | 0.1699 | 0.1540 | 0.4043 | 0.4161 |
| MD STD(Cells centroid) | 0.0233 | 0.0548 | 0.1147 | 0.1650 |
| MD STD(Junctions) | 0.0405 | 0.0547 | 0.0912 | 0.1499 |
| MD STD(Wing outlines) | 0.1336 | 0.1708 | 0.3651 | 0.3443 |
| Subtract value (Cells) | 0.0000 | 6.0000 | 10.0000 | 53.0000 |
| Subtract value (Junctions) | 0.0000 | 2.0000 | 7.0000 | 22.0000 |
| Wings area differences | 0.4228 | 0.1507 | 1.1724 | 9.9337 |

Table S2: The outcome of measuring the overall geometric features extracted by WingAnalogy for the wings.

| | *Apis mellifera* | | *Ischnura elegans* | | *Crocothemis erythraea* | | *Schistocerca gregaria* | |
|---|---|---|---|---|---|---|---|---|
| | left | right | left | right | left | right | left | right |
| Wing area ($mm^2$) | 17.4124 | 17.8352 | 33.1788 | 33.0280 | 227.8660 | 229.0385 | 464.8849 | 454.9512 |
| Wing length ($mm$) | 9.1468 | 9.1355 | 13.0947 | 13.1151 | 34.1897 | 34.1963 | 36.8002 | 37.3204 |
| Wing width ($mm$) | 1.9037 | 1.9523 | 2.5338 | 2.5183 | 6.6647 | 6.6977 | 12.6327 | 12.1904 |
| Wing perimeter ($mm$) | 20.2807 | 20.4033 | 28.7270 | 28.8562 | 74.8585 | 74.9240 | 103.2428 | 105.6995 |
| Number of cells | 13 | 13 | 140 | 142 | 447 | 440 | 645 | 667 |
| Number of junctions | 24 | 24 | 212 | 218 | 825 | 815 | 1159 | 1212 |

Table S3: Data of Figure 3

| | | *Apis mellifera* | *Ischnura elegans* | *Crocothemis erythraea* | *Schistocerca gregaria* |
|---|---|---|---|---|---|
| Set 1 | Regression(Area) | 0.8291 | 0.9899 | 0.9173 | 0.7659 |
| | Regression(Length) | 0.9945 | 0.9987 | 0.9002 | 0.7683 |
| | Regression(Width) | 0.5212 | 0.9848 | 0.9461 | 0.7315 |
| | Regression(Circularity) | 0.3198 | 0.9915 | 0.9283 | 0.2647 |
| | NRMSE(Area) | 0.8752 | 0.1568 | 0.4020 | 0.7262 |
| | NRMSE(Length) | 0.1127 | 0.0604 | 0.4424 | 0.6976 |
| | NRMSE(Width) | 1.3139 | 0.1817 | 0.3316 | 0.7894 |
| | NRMSE(Circularity) | 1.0096 | 0.1321 | 0.3898 | 1.6260 |
| | MD(Centroid) | 0.0554 | 0.0500 | 0.1677 | 0.3439 |
| | MD STD(Centroid) | 0.0419 | 0.0383 | 0.1540 | 0.1770 |
| Set 2 | Regression(Area) | 0.9996 | 0.9890 | 0.7982 | 0.6977 |
| | Regression(Length) | 0.9984 | 0.9676 | 0.9551 | 0.6748 |
| | Regression(Width) | 0.9987 | 0.9926 | 0.7623 | 0.7518 |
| | Regression(Circularity) | 0.9589 | 0.9740 | 0.9183 | 0.6428 |
| | NRMSE(Area) | 0.2363 | 0.1546 | 0.6473 | 0.8479 |
| | NRMSE(Length) | 0.2626 | 0.2608 | 0.3025 | 0.8583 |
| | NRMSE(Width) | 0.1764 | 0.1278 | 0.6713 | 0.7554 |
| | NRMSE(Circularity) | 0.2997 | 0.2587 | 0.3976 | 0.9174 |
| | MD(Centroid) | 0.0489 | 0.0638 | 0.2111 | 0.3577 |
| | MD STD(Centroid) | 0.0185 | 0.0564 | 0.1421 | 0.1620 |
| Set 3 | Regression(Area) | 1.0000 | 0.9738 | 0.8492 | 0.4716 |
| | Regression(Length) | 0.9980 | 0.8582 | 0.9050 | 0.5093 |
| | Regression(Width) | 0.9987 | 0.9893 | 0.5706 | 0.5575 |
| | Regression(Circularity) | 0.9840 | 0.6899 | 0.8203 | 0.5192 |
| | NRMSE(Area) | 0.0696 | 0.2453 | 0.5617 | 1.1361 |
| | NRMSE(Length) | 0.1343 | 0.5296 | 0.4470 | 0.9803 |
| | NRMSE(Width) | 0.0693 | 0.1627 | 0.9151 | 1.0020 |
| | NRMSE(Circularity) | 0.2269 | 0.7464 | 0.5875 | 0.9560 |
| | MD(Centroid) | 0.0390 | 0.0834 | 0.2100 | 0.3446 |
| | MD STD(Centroid) | 0.0135 | 0.0548 | 0.1203 | 0.1553 |
| Set 4 | Regression(Area) | 1.0000 | 0.9660 | 0.8658 | 0.6779 |
| | Regression(Length) | 1.0000 | 0.8645 | 0.8590 | 0.6290 |
| | Regression(Width) | 1.0000 | 0.9735 | 0.7853 | 0.6324 |
| | Regression(Circularity) | 1.0000 | 0.8754 | 0.7185 | 0.4014 |
| | NRMSE(Area) | 0.3135 | 0.3175 | 0.5447 | 0.8331 |
| | NRMSE(Length) | 0.1159 | 0.5754 | 0.5567 | 0.8497 |
| | NRMSE(Width) | 0.9568 | 0.2570 | 0.6708 | 0.8650 |
| | NRMSE(Circularity) | 0.3080 | 0.6397 | 0.7501 | 1.0744 |
| | MD(Centroid) | 0.0433 | 0.0615 | 0.1902 | 0.3005 |
| | MD STD(Centroid) | 0.0355 | 0.0467 | 0.1038 | 0.1415 |
| Set 5 | Regression(Area) | 1.0000 | 0.9561 | 0.9438 | 0.5952 |
| | Regression(Length) | 1.0000 | 0.8927 | 0.9324 | 0.6642 |
| | Regression(Width) | 1.0000 | 0.9276 | 0.8367 | 0.6283 |
| | Regression(Circularity)- | 1.0000 | 0.4067 | 0.7519 | 0.5356 |
| | NRMSE(Area) | 0.2345 | 0.3244 | 0.3396 | 0.8209 |
| | NRMSE(Length) | 0.2597 | 0.5042 | 0.3687 | 0.7880 |
| | NRMSE(Width) | 0.2176 | 0.3943 | 0.5740 | 0.8141 |
| | NRMSE(Circularity) | 0.5245 | 1.1025 | 0.6909 | 0.9612 |
| | MD(Centroid) | 0.0352 | 0.0934 | 0.1538 | 0.2154 |
| | MD STD(Centroid) | 0.0021 | 0.0511 | 0.0801 | 0.1140 |

Table S4: This table compares measurements obtained for the right wing in Figure S13, including area, length, and circularity, using both WingAnalogy and ImageJ.

| | Measured by WingAnalogy | | | Measured by ImageJ | | | Accuracy % | | |
|---|---|---|---|---|---|---|---|---|---|
| Cells labels | Area | Length | Circularity | Area | Length | Circularity | Area | Length | Circularity |
| 1 | 6345 | 268.27 | 0.23 | 6348 | 266.71 | 0.24 | 0.05 | 0.58 | 4.17 |
| 2 | 1547 | 81.74 | 0.53 | 1547 | 79.46 | 0.57 | 0.00 | 2.87 | 7.02 |
| 3 | 5540 | 235.6 | 0.26 | 5543 | 234.03 | 0.27 | 0.05 | 0.67 | 3.70 |
| 4 | 3561 | 141.17 | 0.42 | 3563 | 140.01 | 0.44 | 0.06 | 0.83 | 4.55 |
| 5 | 2097 | 106.25 | 0.43 | 2100 | 103.92 | 0.45 | 0.14 | 2.24 | 4.44 |
| 6 | 2115 | 111.61 | 0.42 | 2116 | 108.63 | 0.45 | 0.05 | 2.74 | 6.67 |
| 7 | 11343 | 199.46 | 0.55 | 11347 | 199.74 | 0.57 | 0.04 | 0.14 | 3.51 |
| 8 | 2876 | 178.84 | 0.21 | 2878 | 174.44 | 0.22 | 0.07 | 2.52 | 4.55 |
| 9 | 5757 | 141.03 | 0.53 | 5760 | 137.06 | 0.55 | 0.05 | 2.90 | 3.64 |
| 10 | 7558 | 160.38 | 0.6 | 7564 | 159.7 | 0.62 | 0.08 | 0.43 | 3.23 |
| 11 | 8302 | 157.28 | 0.65 | 8303 | 154.11 | 0.67 | 0.01 | 2.06 | 2.99 |
| 12 | 5071 | 304.36 | 0.14 | 5076 | 301.94 | 0.15 | 0.10 | 0.80 | 6.67 |
| 13 | 9659 | 196.87 | 0.56 | 9662 | 193.3 | 0.58 | 0.03 | 1.85 | 3.45 |
| Average | | | | | | | 0.056 | 1.59 | 3.7 |

Table S5: This table compares measurements obtained for the left wing in Figure S13, including area, length, and circularity, using both WingAnalogy and ImageJ.

| | Measured by WingAnalogy | | | Measured by ImageJ | | | Accuracy % | | |
|---|---|---|---|---|---|---|---|---|---|
| Cells labels | Area | Length | Circularity | Area | Length | Circularity | Area | Length | Circularity |
| 1 | 5906 | 257.381 | 0.237 | 5917 | 254.386 | 0.243 | 0.19 | 1.18 | 2.47 |
| 2 | 1466 | 76.485 | 0.547 | 1467 | 74.888 | 0.591 | 0.07 | 2.13 | 7.45 |
| 3 | 4781 | 227.721 | 0.241 | 4787 | 225.354 | 0.248 | 0.13 | 1.05 | 2.82 |
| 4 | 3301 | 130.465 | 0.448 | 3308 | 129.062 | 0.463 | 0.21 | 1.09 | 3.24 |
| 5 | 1957 | 104.676 | 0.408 | 1958 | 101.056 | 0.432 | 0.05 | 3.58 | 5.56 |
| 6 | 2127 | 116.017 | 0.39 | 2127 | 113.407 | 0.414 | 0.00 | 2.30 | 5.80 |
| 7 | 11424 | 199.812 | 0.51 | 11427 | 198.001 | 0.527 | 0.03 | 0.91 | 3.23 |
| 8 | 2191 | 180.712 | 0.167 | 2197 | 178.987 | 0.172 | 0.27 | 0.96 | 2.91 |
| 9 | 5100 | 134.648 | 0.503 | 5105 | 130.638 | 0.521 | 0.10 | 3.07 | 3.45 |
| 10 | 7334 | 159.38 | 0.581 | 7340 | 157.156 | 0.606 | 0.08 | 1.42 | 4.13 |
| 11 | 7806 | 149.777 | 0.662 | 7808 | 147.363 | 0.689 | 0.03 | 1.64 | 3.92 |
| 12 | 7104 | 306.232 | 0.197 | 7106 | 304.002 | 0.201 | 0.03 | 0.73 | 1.99 |
| 13 | 9386 | 188.82 | 0.573 | 9393 | 187.211 | 0.589 | 0.07 | 0.86 | 2.72 |
| Average | | | | | | | 0.096 | 1.6 | 3.8 |

Table S6: This table compares measurements obtained for the left wing in Figure S13, including area, length, and circularity, using both WingAnalogy and ImageJ.

| | | WingAnalogy | ImageJ | Accuracy % |
|---|---|---|---|---|
| Regression | Area | 0.9743 | 0.9744 | 0.01 |
| | Length | 0.9969 | 0.9965 | 0.04 |
| | Circulratiy | 0.9838 | 0.9848 | 0.1 |
| NRMSE | Area | 0.2305 | 0.2298 | 0.3 |
| | Length | 0.1014 | 0.1054 | 3.8 |
| | Circulratiy | 0.1851 | 0.1787 | 3.6 |

# 4 Supplementary Codes

## 4.1 Code S1: Get Image

Code S1 is the function developed for importing the wing image. Line 1 in Code S1 defines the function.

**Input.** The input for this function is not explicitly defined. To import the wing image, we utilized the *uigetfile* function in MATLAB, allowing the user to import a desired file (in this case, the wing image). The output of *uigetfile* is the 'file' name and the 'path' where the file is stored.
**Ouput.** The outputs of this function is as follows:

- *imageBinaryImage*. This variable stores the binarized image of the wing.
- *imageOrgImage*. This variable stores the original matrix of the imported wing image.
- *imageWidth*. This variable stores the width of the imported image.
- *imageHeight*. This variable stores the height of the imported image.

**Code Description.** This code reads the image matrix in line 5. Line 6 checks whether the image is in RGB format. If the image is in RGB, the *rgb2gray* function in line 7 converts the image to grayscale. Line 9 is included to binarize the image using the *imbinarize* function. This function is a built-in MATLAB function that utilizes Otsu's method. The function requires a threshold value, and we have set it to 0.54 in this code. Lines 10 to 14 handle the cropping of the image to fit within a frame.

Code S1: GetImage code in MATLAB, for manual importing of the wing Image

```
1  function [imageBinaryImage,imageOrgImage,imageWidth,imageHeight]=GetImage
2  [file,path,indx] = uigetfile( '*.*');
3  ThresholdEditField = 0.54;
4  if indx
5      imageOrgImage = imread([path file]);
6      if size(imageOrgImage,3)==3
7          imageOrgImage = rgb2gray(imageOrgImage);
8      end
9      imageBinaryImage= imbinarize(imageOrgImage,ThresholdEditField); %Otsu's Method
10     [r,c] = find(imageBinaryImage==0);
11     imageBinaryImage = imageBinaryImage(min(min(r))-3:max(max(r))+3,min(min(c))-3:max(max(c))+3);
12    imageOrgImage = imageOrgImage(min(min(r))-3:max(max(r))+3,min(min(c))-3:max(max(c))+3);
13     imageWidth = size(imageOrgImage,2);
14     imageHeight = size(imageOrgImage,1);
15 end
16 end
```

## 4.2 Code S2: Region Growing

Region growing, as introduced in the main manuscript, extracts the boundary of a closed domain surrounded by black pixels.

**Inputs.** Three inputs are defined for Code S2:

- *imageBinaryImage*. The binary image, the first output of Code S1.
- *r*. The row number (y-coordinate) of a random seed inside a desired region.
- *c*. The column number (x-coordinate) of a random seed inside a desired region.

**Outputs.** Code S2 has four outputs.

- *cell_whiteID*. An N-by-2 array storing the coordinates of detected white pixels inside the desired region is used to generate the corresponding matrix for the area, length, and circularity distribution of wing cells.
- *n_white*. The number of detected white pixels in the desired region represents the area of the detected region.
- *im*. The image after detection of the region. When a desired region in the wing is detected, its color changes to gray to avoid repeated detection.
- *boundary*. An N-by-2 array containing the coordinates of the detected region.

**Code Description:**

- **line 3.** The binary image's matrix is converted to double because, upon importing an image into the function, the color of the detected area changes to gray, which is not possible in a binary image.
- **Line 4.** *FWP* represents 'Found White Pixels'. Initially, $r$ and $c$ (the second and third inputs of Code S2) go into *FWP*. This variable updates during the algorithm's execution.
- **Lines 5-6.** *border* and *cell_whiteID* are empty matrices that store the coordinates of detected boundary pixels and white pixels inside the region, respectively.
- **Lines 7-34.** The while loop starts from the initial seed point ($r$, $c$) and checks for new white (lines 8-18) and black pixels (lines 19-30) around the seed pixel. Each time it detects a new white or black pixel, it saves their coordinates separately. Also, each time it finds white pixels, it changes the color of those detected pixels and continues checking for new white pixels around those detected points. The while loop continues until there are still white pixels inside the region.
- **Lines 35-59.** In the while loop, detected pixels on the boundary are not in order. It means that the coordinates don't define a closed loop, and they are only points on the boundary that are stored irregularly. In lines 35-59, the code constructs a closed loop of those extracted points on the boundary of the domain.

Code S2: Region Growing Code in MATLAB

```
1  function [cell_whiteID,n_white,im,boundary]= RegionGrowing(imageBinaryImage,r,c)
2  %r, and c represent the row and column number of a random seed inside the desired
       region
3  im = double(imageBinaryImage);
4  FWP=[c,r];       % FWP:found white pixel(first white pixel is choosen by user)
5  border=[];
6  cell_whiteID=[];
7  while ~isempty(FWP) %the code continues while it does not find new white pixe
8      X=[FWP(:,1) FWP(:,1)      FWP(:,1)+1      FWP(:,1)      FWP(:,1)-1];
9      Y=[FWP(:,2) FWP(:,2)-1      FWP(:,2)      FWP(:,2)+1      FWP(:,2)];
10     X=X(:); Y=Y(:);
11     l=length(X);
12     new_FWP=[];
13     for i=1:l
14         if im(Y(i),X(i))==1
15             im(Y(i),X(i))=0.8; % changes the color of found white pixel to bright
                   grey
16             new_FWP=[new_FWP; X(i) Y(i)];   %#ok %new_FWP: new Found White Pixel
17         end
18     end
19     % Finding new black pixels in the neighbor of FWP on 8 directions
```

```
    X=[FWP(:,1) FWP(:,1)+1 FWP(:,1) FWP(:,1)-1 FWP(:,1)-1 FWP(:,1)-1 FWP(:,1)+1 FWP(:,1)+1];
    Y=[FWP(:,2)-1 FWP(:,2) FWP(:,2)+1 FWP(:,2) FWP(:,2)-1 FWP(:,2)+1 FWP(:,2)-1 FWP(:,2)+1];
    X=X(:); Y=Y(:);
    l=length(X);
    new_FBP=[];
    for i=1:l
        if im(Y(i),X(i))==0
            im(Y(i),X(i))=0.1; % changes the color of found black pixel to dark grey
            new_FBP=[new_FBP; X(i) Y(i)];    %#ok %new_FBP: new Found Black Pixel
        end
    end
    border=[border;new_FBP];                              %#ok
    cell_whiteID=[cell_whiteID;new_FWP];    %#ok
    FWP=new_FWP;
end
border = unique(border,'rows');
n_white=size(unique(cell_whiteID,'rows'),1);
im_bw = zeros(size(im,1),size(im,2));
for i=1:size(border,1)
    im_bw(border(i,2),border(i,1))=1;
    im(border(i,2),border(i,1))=0;
end
BW = imbinarize(im_bw);
try
    boundary = bwtraceboundary(BW , [border(i,2),border(i,1)],'S');
catch
    try
        boundary = bwtraceboundary(BW , [border(i,2),border(i,1)],'E');
    catch
        try
            boundary = bwtraceboundary(BW , [border(i,2),border(i,1)],'N');
        catch
            boundary = bwtraceboundary(BW , [border(i,2),border(i,1)],'W');
        end
    end
end
boundary = [boundary(:,2) boundary(:,1)];
boundary(:,2)=size(im,1)-boundary(:,2);
[boundary(:,1), boundary(:,2)] = poly2cw(boundary(:,1), boundary(:,2));
im = double(im);
end
```

## 4.3 Code S3: Wing Cell Segmentation

This code is developed to segment all cells of a wing by repeatedly applying the *Region_Growing* function.
**Input.** The only input of this function is the binary image generated by the *GetImage* function.
**Outputs.** Code S3 has six outputs as follows.

- *ContourMatrix_Area.* Stores the matrix generated by *RegionGrowing* that contains the corresponding matrix for the distribution of wing cells' areas.
- *ContourMatrix_Length.* Similar to the previous one, this variable contains the distribution of wing cells' lengths.
- *ContourMatrix_Circularity.* This variable similarly contains the distribution of wing cells' circularities.
- *wingOutline.* This is an N-by-2 array that contains the coordinates of the wing outline, extracted from *RegionGrowing.*
- *wingCells.* This is a one-by-N cell array. The number of cells in this array represents the number of wing cells, and each cell array contains the coordinates of one wing cell boundary.
- *wingInfo.* This is an N-by-6 matrix. N represents the number of cells. Each row of this variable has six columns representing the cell index, the x-coordinate of the cell centroid, the y-coordinate of the cell centroid, the cell's area, the cell's length, and the cell's circularity.

**Code Description.**

- **Lines 8-11.** These lines generate a gray frame around the image. This is necessary for extracting the outline of the wing. Otherwise, the *RegionGrowing* function faces problems when it reaches the edge of the image (first and last columns and rows of the image matrix).
- **Line 16.** *RegionGrowing* runs in this line to extract the outline of the wing.
- **Lines 32-63.** In line 32, a *while* loop starts. In this while loop, the *RegionGrowing* function runs recursively until all cells are detected. As mentioned, the second and third inputs of the *RegionGrowing* function are one seed point inside the region. Keep in mind that the outline is already detected, and as a result, the color of the outer region of the wing image is already gray. To find the first seed inside the first cell, in line 30 the code finds all white pixels, and in line 33, it uses only the first detected white pixel. As the outer region of the wing is gray, this white pixel is located inside one of the cells. Then, *RegionGrowing* extracts that specific cell's boundary and changes the color of that cell to gray. Then inside the loop in line 60, the code again searches for new white pixels, and absolutely these white pixels are inside not detected cells because all detected cells have changed color to gray. This while loop continues until the code finds no new white pixels. Video S3 in the supplementary materials visually demonstrates this procedure.

Code S3: Wing Cells Segmentation Code in MATLAB

```
1  function [ContourMatrix_Area,...
2      ContourMatrix_Length,...
3      ContourMatrix_Circularity,...
4      wingOutline,...
5      wingCells,...
6      wingInfo] = cellSegment(imageBinaryImage)
7  w = double(imageBinaryImage);
8  w(1,:)=.5;
9  w(end,:)=.5;
10 w(:,1)=.5;
11 w(:,end)=0.5;
12 s_im=size(w);
13 r=2; c=2;
14 wingInfo = [];
15 wingCells ={};
16 [cell_whiteID,~,w,boundary]=RegionGrowing(w,c,r);
17 wingOutline= boundary;
18 ContourMatrix_Area=zeros(s_im(1),s_im(2));
19 for i=1:size(cell_whiteID,1)
20     ContourMatrix_Area(cell_whiteID(i,2),cell_whiteID(i,1))=NaN;
21 end
22 ContourMatrix_Length=zeros(s_im(1),s_im(2));
23 for i=1:size(cell_whiteID,1)
24     ContourMatrix_Length(cell_whiteID(i,2),cell_whiteID(i,1))=NaN;
```

```
end
ContourMatrix_Circularity=zeros(s_im(1),s_im(2));
for i=1:size(cell_whiteID,1)
    ContourMatrix_Circularity(cell_whiteID(i,2),cell_whiteID(i,1))=NaN;
end
[r,c]=find(w==1);
count=0;
allDetected = 0;
while sum(r)>0    [cell_whiteID,n_white,w,boundary]=RegionGrowing(w,r(1),c(1));
    if n_white > 8
        wingCells{end+1}= boundary;
        count=count+1;
        s_b=size(boundary,1);
        all_l=[];
        for i=1:s_b
            l=sqrt(((boundary(i,1)-boundary(:,1)).^2)+(boundary(i,2)-boundary(:,2)).^2);
            all_l=[all_l;l];  %#ok
        end
        all_ll = [];
        for i=1:size(boundary,1)-1
            ll=sqrt(((boundary(i,1)-boundary(i+1,1)).^2)+(boundary(i,2)-boundary(i+1,2)).^2);
            all_ll=[all_ll;ll];  %#ok
        end
        Perimeter = sum(all_ll);
        Rc = Perimeter/(2*pi);
        for i=1:size(cell_whiteID,1)
            if ~isempty(all_l)
                ContourMatrix_Area(cell_whiteID(i,2),cell_whiteID(i,1))=n_white;
                ContourMatrix_Length(cell_whiteID(i,2),cell_whiteID(i,1))=max(all_l);
                ContourMatrix_Circularity(cell_whiteID(i,2),cell_whiteID(i,1)) = n_white/(pi*(Rc^2));
            end
        end
        wingInfo(count,:) = [count...
        mean(boundary(:,1))...
        mean(boundary(:,2))...
        n_white...
        max(all_l)...
        n_white/(pi*(Rc^2))];
    end
    [r,c]=find(w==1);
    id_gray = w==0.1;
    w(id_gray) = 0;
end
id = ContourMatrix_Area == 0;
ContourMatrix_Area(id) = nan;
ContourMatrix_Area = ContourMatrix_Area(end:-1:1,:);
id= ContourMatrix_Length == 0;
ContourMatrix_Length(id) = nan;
ContourMatrix_Length = ContourMatrix_Length(end:-1:1,:);
id= ContourMatrix_Circularity == 0;
ContourMatrix_Circularity(id) = nan;
ContourMatrix_Circularity = ContourMatrix_Circularity(end:-1:1,:);
end
```

## 4.4 Code S4: Junction Detection/Skeletonization

This function is developed to detect junctions of the wing.
**Input.** The only input for this function is the binary image of the wing.
**Outputs.** This function has two outputs:

- *junctions.* A structure variable with the following fields.
  - *junctions.c.* An N-by-1 array storing the X coordinate of junctions.
  - *junctions.r.* An N-by-1 array storing the Y coordinate of junctions.
  - *junctions.surround.x.* An N-by-20 array containing the X coordinate of a circle with a radius of 1.5 pixels around junctions (used in the detection of veins).
  - *junctions.surround.y.* An N-by-20 array containing the Y coordinate of a circle with a radius of 1.5 pixels around junctions (used in the detection of veins).

  N in this array represents the number of detected junctions.
- *skeletonizedImage.* A matrix representing the skeletonized image of the wing.

**Code Description.**

- **Lines 5-11.** These lines apply the distance transform on the wing image. This feature can be turned on or off by setting the value of the *Distancetransform* variable to 1 or 0. Distance transform can detect the location of the Pterostigma in the wing.
- **Line 14.** Applies the thinning method to generate the skeletonized image.
- **Lines 17-40.** Implements the conditions used to detect the locations of junctions.
- **Lines 44-52.** Modifies detected junctions to remove junctions located within a distance of less than 3 pixels from each other.

Code S4: Junction Detection code in MATLAB, for automated Wing Junction Detection

```
1 function [junctions, skeletonizedImage]=junctionDetection(imageBinaryImage)
2 wOrg = imageBinaryImage;
3 w = imageBinaryImage;
4 Distancetransform = 0; %This value can be 0 or 1
5 if Distancetransform
6     w = bwdist(w);
7     w = w/max(max(w));
8     w(w>0.7) =1;
9     w(w<=0.7) = 0;
10    w=wOrg + w;
11 end
12 w=~w;
13 w = double(w);
14 w = bwmorph(w, 'thin',100);
15 w = double(w);
16 skeletonizedImage = ~w;
17 w1 = w(2:end-1,2:end-1);
18 w2 = w(1:end-2,2:end-1);
19 w3 = w(1:end-2,3:end);
20 w4 = w(2:end-1,3:end);
21 w5 = w(3:end,3:end);
22 w6 = w(3:end,2:end-1);
23 w7 = w(3:end,1:end-2);
24 w8 = w(2:end-1,1:end-2);
25 w9 = w(1:end-2,1:end-2);
26 id1 = w1+w2+w4+w6+w8==4 & w1==1;
27 id2 = w1+w2+w4+w6+w8==5  & w1==1;
28 id3 =(w1+w2+w4+w7)==4  & w1==1;
29 id4 = (w1+w4+w6+w9)==4  & w1==1;
30 id5 = (w1+w6+w8+w3)==4  & w1==1;
31 id6 = (w1+w2+w8+w5)==4  & w1==1;
32 id7 = (w1+w3+w5+w8-w4)==4  & w1==1;
```

```
id8 = (w1+w5+w7+w2-w6)==4  & w1==1;
id9 = (w1+w7+w9+w4-w8)==4  & w1==1;
id10 = (w1+w9+w3+w6-w2)==4  & w1==1;
id11 = (w1+w3+w5+w7+w9)==4  & w1==1;
id12 = (w1+w3+w5+w7+w9)==5  & w1==1;
id13 = w2+w3+w4+w5+w6+w7+w8+w9==1 & w1==1;
id = id1+id2+id3+id4+id5+id6+id7+id8+id9+id10+id11+id12+id13;
[r,c] = find(id>=1);
r=r+1;
c=c+1;
finalJoints=[];
while ~isempty(r)
    rCP = r(1);
    cCP = c(1);
    d = sqrt((r-rCP).^2+(c-cCP).^2);
    id = d<=3;
    finalJoints = [finalJoints; mean(r(id)) mean(c(id))];   %#ok
    r(id)=[];
    c(id)=[];
end
finalJoints = round(unique(finalJoints,'rows'));
junctions.c =  finalJoints(:,2);
junctions.r =  finalJoints(:,1);
t = linspace(0,2*pi,20);
x = 1.5*cos(t);
y = 1.5*sin(t);
junctions.surround.x = zeros(length(junctions.c),20);
junctions.surround.y = zeros(length(junctions.r),20);
for i = 1 : length(junctions.c)
    junctions.surround.x(i,:)= x+ junctions.c(i);
    junctions.surround.y(i,:) = y+ junctions.r(i);
end
end
```

### 4.5 Code S5: Automated Superimposition Using Particle Swarm Optimization (PSO)

This code is developed for automated superimposing wings. The code consists of four functions.

1. ***PSO_Superimposition.*** This is the main function of the PSO algorithm specifically developed for automated superimposing.

   **Inputs.** This function has two inputs.

   - *outline_im1.* This variable refers to the extracted outline from the first image.
   - *outline_im2.* This variable refers to the extracted outline from the second image.

   **Output**. *BestParticle* is the only output of this function. It is a 1-by-3 array in which the first and second elements refer to the x and y coordinates of the translation of the first image, and the third element relates to the rotation of the second.

   **Code Description.**

   - **Lines 2-6.** The number of variables for each particle in the PSO algorithm and their boundary conditions are defined in these lines. Each particle consists of three variables. The first and second refer to the x and y coordinates for translating the wing. The third one refers to the angle of wing rotation.
   - **Lines7-35.** PSO parameters are defined in these lines. The number of particles is defined in Line 9, and the maximum number of iterations is defined in Line 8. Besides, the initial particles are defined in the for loop in Lines 23-35.
   - **Lines 36-57:** These lines are the main loop of the PSO.

2. ***costFun.*** This function is called in lines 27 and 46 of the *PSO_Superimposition* function. It calculates the distance between wing outlines following the repositioning of the second image, considering the translation and rotation of an individual particle. A penalty function in Lines 74-76 is the penalty for an individual that induces an angle of more than 10 degrees between two wings.

3. ***distanceFun.*** This function is called in Line 69 of the *costFun* to calculate the distance between the first wing and the second wing after translation and rotation.

4. ***wingsAngle.*** This function is called in Lines 72 and 73 to measure the angle of each wing concerning the horizontal axis line.

Code S5: Particle Swarm Optimization Code Developed for Wings Automated Superimposition

```
1  function BestParticle=PSO_Superimposition(outline_im1,outline_im2)
2  %% Problem Definition
3  nVar = 3;
4  varSize = [1 nVar];
5  varMin  = [-10 -10  -90];
6  varMax =  [ 10  10   90];
7  %% PSO Parameters
8  maxIt = 40;
9  nParticle = 30;
10 w = 1;
11 wDamp = 0.95;
12 c1 = 1.2;
13 c2 = 1.2;
14 %% Initialization
15 Particle.Position = zeros(varSize);
16 Particle.Cost = [];
17 Particle.Velocity = [];
18 Particle.Best.Position =  [];
19 Particle.Best.Cost = [];
20 Particle = repmat(Particle,nParticle,1);
21 gBest.Cost = -inf;
22 gBest.Position = zeros(varSize);
23 for i = 1 : nParticle
24     for j = 1 : nVar
25         Particle(i).Position(1,j) = unifrnd(varMin(j),varMax(j),1);
26     end
27     Particle(i).Cost = costFun(Particle(i).Position, outline_im1, outline_im2);
28     Particle(i).Velocity = zeros(varSize);
```

```
    Particle(i).Best.Position = Particle(i).Position;
    Particle(i).Best.Cost = Particle(i).Cost;
    if Particle(i).Best.Cost > gBest.Cost
        gBest.Cost = Particle(i).Best.Cost;
        gBest.Position = Particle(i).Best.Position;
    end
end
%% Main Loop
for j = 1 : maxIt
    for i=1:nParticle
        Particle(i).Velocity  = w*Particle(i).Velocity ...
            + rand(varSize)*c1.*(Particle(i).Best.Position- Particle(i).Position) ...
            + rand(varSize)*c2.*(gBest.Position-Particle(i).Position);
        Particle(i).Position = Particle(i).Position+Particle(i).Velocity;
        id1= Particle(i).Position > varMax;
        id2= Particle(i).Position < varMin;
        Particle(i).Position(id1) = varMax(id1);
        Particle(i).Cost = costFun(Particle(i).Position, outline_im1, outline_im2);
        if Particle(i).Cost> Particle(i).Best.Cost
            Particle(i).Best.Cost = Particle(i).Cost;
            Particle(i).Best.Position = Particle(i).Position;
        end
        if Particle(i).Best.Cost > gBest.Cost
            gBest.Cost = Particle(i).Best.Cost;
            gBest.Position = Particle(i).Best.Position;
        end
    end
    w = w*wDamp;
end
BestParticle = gBest.Position;
end
function y = costFun(indParticle,...
   outline_im1,...
   outline_im2)
Xc = indParticle(1);
Yc = indParticle(2);
Alpha = indParticle(3);
rotateMatrix = @(t) ([cosd(t) -sind(t) ; sind(t) cosd(t)]);
outline_im2_PSO = [outline_im2(:,1)-Xc    outline_im2(:,2)-Yc];
outline_im2_PSO = (rotateMatrix(-Alpha)*outline_im2_PSO')';
y = distanceFun(outline_im1,outline_im2_PSO);
x1 = outline_im2_PSO(:,1);
y1 = outline_im2_PSO(:,2);
teta2 = wingsAngle(x1,y1);
teta1 = wingsAngle(outline_im1(:,1),outline_im1(:,1));
if  abs(abs(teta1)-abs(teta2))>10
    y= -inf;
end
end
function d = distanceFun(outline_im1,outline_im2_PSO)
x1 = outline_im1(:,1);
y1 =outline_im1(:,2);
x2 = outline_im2_PSO(:,1);
y2 = outline_im2_PSO(:,2);
[X1,X2] = meshgrid(x1,x2);
[Y1,Y2] = meshgrid(y1,y2);
d = -mean(min(sqrt(((X1-X2).^2)+((Y1-Y2).^2))));
end
function teta = wingsAngle(x,y)
d_old=0;
for i = 1 : length(x)
```

```
    id1= i ;
    [d, id2] = max(sqrt(  ((x(i) - x).^2) + ((y(i)-y).^2)  ));
    if d>d_old
        lineID = [id1 id2];
    end
end
teta = atand((y(lineID(2))-y(lineID(1)))/(x(lineID(2))-x(lineID(1))));
end
```

### 4.6 Code S6: Computing Asymmetry

This function is developed for computing the asymmetry of two wings.
**Inputs.** Code S6 requires 18 inputs.

- *image1_cellsCentroid.* An N-by-2 array contains the x and y coordinates of the cell centroid coordinates of the first wing. N refers to the number of cells in the first image.
- *image2_cellsCentroid.* An N-by-2 array contains the x and y coordinates of the cell centroid coordinates of the second wing. N refers to the number of cells in the second image.
- *image1_cellsArea.* An N-by-1 array containing the area of cells in the first image. N refers to the number of cells in the first image.
- *image2_cellsArea.* An N-by-1 array containing the area of cells in the second image. N refers to the number of cells in the second image.
- *image1_cellsLength.* An N-by-1 array containing the length of cells in the first image. N refers to the number of cells in the first image.
- *image2_cellsLength.* An N-by-1 array containing the length of cells in the second image. N refers to the number of cells in the second image.
- *image1_cellsWidth.* An N-by-1 array containing the width of cells in the first image. N refers to the number of cells in the first image.
- *image2_cellsWidth.* An N-by-1 array containing the width of cells in the second image. N refers to the number of cells in the second image.
- *image1_cellsCircularity.* An N-by-1 array containing the circularity of cells in the first image. N refers to the number of cells in the first image.
- *image2_cellsCircularity.* An N-by-1 array containing the circularity of cells in the second image. N refers to the number of cells in the second image.
- *image1_junctionsAmount.* Number of junctions in the first image.
- *image2_junctionsAmount.* Number of junctions in the second image.
- *image1_cellsAmount.* Number of cells in the first image.
- *image2_cellsAmount.* Number of cells in the second image.
- *image1_junctionsCoordinate.* An N-by-2 array containing the coordinate of junctions in the first image. N refers to the number of junctions in the first image.
- *image2_junctionsCoordinate.* An N-by-2 array containing the coordinate of junctions in the second image. N refers to the number of junctions in the second image.
- *image1_outline.* An N-by-2 array containing the coordinate of the first image outline. N refers to the number of points on the outline of the first image.
- *image2_outline.* An N-by-2 array containing the coordinate of the second image outline. N refers to the number of points on the outline of the second image.

**Outputs.** Code S6 generates 16 outputs.

- *Im1VsIm2_cellsCentroidMeanDistance.* The mean distance between corresponding cell centroids of the wings.
- *Im1VsIm2_cellsCentroidMeanDistanceSTD.* The standard deviation of the mean distance between corresponding cell centroids of the wings.
- *Im1VsIm2_NRMSE_cellsArea.* The Normalized Root Mean Square Error of wing cells area.
- *Im1VsIm2_NRMSE_cellsLength.* The Normalized Root Mean Square Error of wing cells length.
- *Im1VsIm2_NRMSE_cellsWidth.* The Normalized Root Mean Square Error of wing cells width.
- *Im1VsIm2_NRMSE_cellsCircularity.* The Normalized Root Mean Square Error of wing cells circularity.
- *Im1AndIm2_junctionsAmountSubtract.* The difference in the number of junctions.
- *Im1AndIm2_cellsAmountSubtract.* The difference in the number of cells.

- *Im1VsIm2_junctionsMeanDistance.* The mean distance between the junctions of wings.
- *Im1VsIm2_junctionsMeanDistanceSTD.* The standard deviation of the mean distances between the junctions of wings.
- *Im1VsIm2_outlineMeanDistance.* the mean distance between the wings outlines.
- *Im1VsIm2_outlineMeanDistanceSTD.* The standard deviation of the mean distance between the wings outlines.
- *Im1VsIm2_Regression_cellsArea.* The regression between the area of the wing cells.
- *Im1VsIm2_Regression_cellsLength.* The regression between the length of the wing cells.
- *Im1VsIm2_Regression_cellsWidth.* The regression between the width of the wing cells.
- *Im1VsIm2_Regression_cellsCircularity.* The regression between the circularity of the wing cells.

**Code Descriptions.**

- **Lines 29-41.** The code in these lines finds the corresponding cells between the first and second images.
- **Line 42.** This line measures the mean distance of corresponding cells.
- **Line 43.** This line measures the standard deviation of the mean distance of corresponding cells.
- **Lines 45-48.** These lines compute the NRMSE between the corresponding cells' area, length, width, and circularity.
- **Line 50.** This line calculates the difference in the number of junctions between two wing images.
- **Line 51.** This line calculates the difference in the number of cells between two wing images.
- **Lines 53-62.** These lines calculate the mean distance of corresponding junctions and their standard deviations.
- **Lines 64-73.** These lines calculate the mean distance between wing outlines and their standard deviations.
- **Lines75-93.** These lines measure the regression of the area, length, width, and circularity of corresponding wing cells.

Code S6: The Developed code for computing the asymmetry of wings

```
1
2  function [Im1VsIm2_cellsCentroidMeanDistance,...
3      Im1VsIm2_cellsCentroidMeanDistanceSTD,...
4      Im1VsIm2_NRMSE_cellsArea,...
5      Im1VsIm2_NRMSE_cellsLength,...
6      Im1VsIm2_NRMSE_cellsWidth,...
7      Im1VsIm2_NRMSE_cellsCircularity,...
8      Im1AndIm2_junctionsAmountSubtract,...
9      Im1AndIm2_cellsAmountSubtract,...
10     Im1VsIm2_junctionsMeanDistance,...
11     Im1VsIm2_junctionsMeanDistanceSTD,...
12     Im1VsIm2_outlineMeanDistance,...
13     Im1VsIm2_outlineMeanDistanceSTD,...
14     Im1VsIm2_Regression_cellsArea,...
15     Im1VsIm2_Regression_cellsLength,...
16     Im1VsIm2_Regression_cellsWidth,...
17     Im1VsIm2_Regression_cellsCircularity]=asymetryComputation(
          image1_cellsCentroid,...
18     image2_cellsCentroid,image1_cellsArea,image2_cellsArea,image1_cellsLength,
          image2_cellsLength,...
19     image1_cellsWidth,image2_cellsWidth,image1_cellsCircularity,
          image2_cellsCircularity,...
20     image1_junctionsAmount,image2_junctionsAmount,image1_cellsAmount,
          image2_cellsAmount,...
21     image1_junctionsCoordinate, image2_junctionsCoordinate,image1_outline,
          image2_outline)
22 % Asymmetry Computation: Image1 Vs Image2
23 Im1VsIm2_Area = zeros(image1_cellsAmount,2);
```

```
Im1VsIm2_Length = zeros(image1_cellsAmount,2);
Im1VsIm2_Width = zeros(image1_cellsAmount,2);
Im1VsIm2_Circularity = zeros(image1_cellsAmount,2);
Im1VsIm2_cellsCentroidDistance = zeros(image1_cellsAmount,1);
Im1VsIm2_correspondingCell = zeros(image1_cellsAmount,2);
for i = 1 : app.image1_cellsAmount
    x = image1_cellsCentroid(i,1);
    y = image1_cellsCentroid(i,2);
    d = sqrt(((x - image2_cellsCentroid(:,1)).^2)+((y - image2_cellsCentroid(:,2)).^2));
    [dMin,id] = min(d);
    j = id(1);
    Im1VsIm2_correspondingCell(i,:) =[i j];
    Im1VsIm2_Area(i,:) = [image1_cellsArea(i)     image2_cellsArea(j)];
    Im1VsIm2_Length(i,:) = [image1_cellsLength(i)   image2_cellsLength(j)];
    Im1VsIm2_Width(i,:) = [image1_cellsWidth(i)    image2_cellsWidth(j)];
    Im1VsIm2_Circularity(i,:) = [image1_cellsCircularity(i)    image2_cellsCircularity(j)];
    Im1VsIm2_cellsCentroidDistance(i) = dMin(1);
end
Im1VsIm2_cellsCentroidMeanDistance = mean(Im1VsIm2_cellsCentroidDistance);
Im1VsIm2_cellsCentroidMeanDistanceSTD = std(Im1VsIm2_cellsCentroidDistance);
%%%%%%%%%%%%%%%%%%%%%%%%%%%%%%%%%%%%%%%%%%%%%%%%%%%%%%%%
Im1VsIm2_NRMSE_cellsArea = goodnessOfFit(Im1VsIm2_Area(:,1),Im1VsIm2_Area(:,2),'NRMSE');
Im1VsIm2_NRMSE_cellsLength = goodnessOfFit(Im1VsIm2_Length(:,1),Im1VsIm2_Length(:,2),'NRMSE');
Im1VsIm2_NRMSE_cellsWidth = goodnessOfFit(Im1VsIm2_Width(:,1),Im1VsIm2_Width(:,2),'NRMSE');
Im1VsIm2_NRMSE_cellsCircularity = goodnessOfFit(Im1VsIm2_Circularity(:,1),Im1VsIm2_Circularity(:,2),'NRMSE');
%%%%%%%%%%%%%%%%%%%%%%%%%%%%%%%%%%%%%%%%%%%%%%%%%%%%%%%%
Im1AndIm2_junctionsAmountSubtract = abs(image1_junctionsAmount - image2_junctionsAmount);
Im1AndIm2_cellsAmountSubtract = abs(image1_cellsAmount - image2_cellsAmount);
%%%%%%%%%%%%%%%%%%%%%%%%%%%%%%%%%%%%%%%%%%%%%%%%%%%%%%%%
Im1VsIm2_junctionsDistance = zeros(image1_junctionsAmount,1);
for i = 1:image1_junctionsAmount
    x = image1_junctionsCoordinate(i,1);
    y = image1_junctionsCoordinate(i,2);
    d = sqrt(((x - image2_junctionsCoordinate(:,1)).^2)+((y - image2_junctionsCoordinate(:,2)).^2));
    dMin = min(min(d));
    Im1VsIm2_junctionsDistance(i) = dMin;
end
Im1VsIm2_junctionsMeanDistance = mean(Im1VsIm2_junctionsDistance);
Im1VsIm2_junctionsMeanDistanceSTD = std(Im1VsIm2_junctionsDistance);
%%%%%%%%%%%%%%%%%%%%%%%%%%%%%%%%%%%%%%%%%%%%%%%%%%%%%%%%
x = image1_outline(:,1);
y = image1_outline(:,2);
Im1VsIm2_outlineDistance = zeros(length(x),1);
for i = 1 : length(x)
    d = sqrt(((x(i) - image2_outline(:,1)).^2)+((y(i) - image2_outline(:,2)).^2));
    dMin = min(min(d));
    Im1VsIm2_outlineDistance(i) = dMin;
end
Im1VsIm2_outlineMeanDistance = mean(Im1VsIm2_outlineDistance);
Im1VsIm2_outlineMeanDistanceSTD = std(Im1VsIm2_outlineDistance);
%%%%%%%%%%%%%%%%%%%%%%%%%%%%%%%%%%%%%%%%%%%%%%%%%%%%%%%%
mdl_area = fitlm(Im1VsIm2_Area(:,1),Im1VsIm2_Area(:,2));
Im1VsIm2_Reg_Area_x = mdl_area.Variables.x1;
Im1VsIm2_Reg_Area_y = mdl_area.Variables.y;
```

```
Im1VsIm2_Regression_cellsArea = regression(Im1VsIm2_Reg_Area_x' ,
    Im1VsIm2_Reg_Area_y');
%%%%%%%%%%%%%%%%%%%%%%%%%%%%%%%%%%%%%%%%%%%%%%%%%%%%%%%%
mdl_length = fitlm(Im1VsIm2_Length(:,1),Im1VsIm2_Length(:,2));
Im1VsIm2_Reg_Length_x = mdl_length.Variables.x1;
Im1VsIm2_Reg_Length_y = mdl_length.Variables.y;
Im1VsIm2_Regression_cellsLength = regression(Im1VsIm2_Reg_Length_x',
    Im1VsIm2_Reg_Length_y');
%%%%%%%%%%%%%%%%%%%%%%%%%%%%%%%%%%%%%%%%%%%%%%%%%%%%%%%%
mdl_width = fitlm(Im1VsIm2_Width(:,1),Im1VsIm2_Width(:,2));
Im1VsIm2_Reg_Width_x = mdl_width.Variables.x1;
Im1VsIm2_Reg_Width_y = mdl_width.Variables.y;
Im1VsIm2_Regression_cellsWidth = regression(Im1VsIm2_Reg_Width_x',
    Im1VsIm2_Reg_Width_y');
%%%%%%%%%%%%%%%%%%%%%%%%%%%%%%%%%%%%%%%%%%%%%%%%%%%%%%%%
mdl_circularity = fitlm(Im1VsIm2_Circularity(:,1),Im1VsIm2_Circularity(:,2));
Im1VsIm2_Reg_Circularity_x = mdl_circularity.Variables.x1;
Im1VsIm2_Reg_Circularity_y = mdl_circularity.Variables.y;
Im1VsIm2_Regression_cellsCircularity = regression(Im1VsIm2_Reg_Circularity_x',
    Im1VsIm2_Reg_Circularity_y');
end
```

# 5 Supplementary Files (Zenodo Repository)

WingAnalogy has generated the following files containing the results of comparing the wings of the honeybee, damselfly, dragonfly, and desert Locust used in this study, along with the overall results for all pairs and their respective sets. All Supporting Data are documented in the Zenodo Repository via the following link, accessible by scanning the QR Code.

https://zenodo.org/doi/10.5281/zenodo.7156257

---

## 5.1 Supplementary Codes

- Desktop App. This folder includes the installable WingSegment.exe file, which is compatible with Windows computers.
- MATLAB App. This folder contains the MATLAB App file for installation as a MATLAB toolbox.
- WingAnalogy Functions. This folder contains all supplementary codes introduced in section 2.

## 5.2 Supplementary Figures

- **Figure S1.** The main window of WingAnalogy.
- **Figure S2.** *New Project* window of WingAnalogy.
- **Figure S3.** *Add/Edit Pair* window of WingAnalogy.
- **Figure S4.** Generated figures by WingAnalogy.
- **Figure S5.** Superimposing tool within the *Add/Edit Pair* Window.
- **Figure S6.** Statistics within *Add/Edit Pair* window.
- **Figure S7.** Histograms within *Add/Edit Pair* window.
- **Figure S8.** Regressions within *Add/Edit Pair* window.
- **Figure S9.** Cell view tool within *Add/Edit Pair* window.
- **Figure S10.** *Local Result* within *Add/Edit Pair* window.
- **Figure S11.** *Local result: Image 1* Window.
- **Figure S12.** Main window of WingAnalogy after defining four pairs.
- **Figure S13.** *All pairs result* window.
- **Figure S14.** Visualization of a honeybee left and right wings with labeled cells.

### 5.3 Supplementary Sample Images

- **Sample Image S1.** *Apis mellifera* (honeybee, Hymenoptera; left forewing).
- **Sample Image S2.** *Apis mellifera* (honeybee, Hymenoptera; right forewing).
- **Sample Image S3.** *Ischnura elegans* (blue-tailed damselfly, Odonata: Zygoptera; left forewing).
- **Sample Image S4.** *Ischnura elegans* (blue-tailed damselfly, Odonata: Zygoptera; right forewing).
- **Sample Image S5.** *Crocothemis erythraea*(Scarlet dragonfly, Odonata:Anisoptera; left forewing)
- **Sample Image S6.** *Crocothemis erythraea*(Scarlet dragonfly, Odonata:Anisoptera; right forewing)
- **Sample Image S7.** *Schistocerca gregaria* (desert locust, Orthoptera; left hindwing)
- **Sample Image S8.** *Schistocerca gregaria* (desert locust, Orthoptera; right hindwing)

### 5.4 Supplementary Videos

- **Video S1.** Preparing image for WingAnalogy.
- **Video S2.** How to define a new project in WinbgAnalogy.
- **Video S3.** How to add a new pair in WingAnalogy.
- **Video S4.** How to use scaling tool in WingAnalogy.
- **Video S5.** How to use superimposing tool in WingAnalogy.
- **Video S6.** Statistics and regressions in WingAnalogy.
- **Video S7.** How to define cell set in WingAnalogy.
- **Video S8.** How to export the result of pair in WingAnalogy.
- **Video S9.** How to save a project in WingAnalogy.
- **Video S10.** How to import a saved project to WingAnalogy.
- **Video S11.** How to export the result of all pairs in WingAnalogy.
- **Video S12.** How to export the result of all sets in WingAnalogy.

### 5.5 Other Supplementary Files

1. Generated reports
   - *Honeybee_GeneratedReport.pdf*
   - *Damselfly_GeneratedReport.pdf*
   - *Dragonfly_GeneratedReport.pdf*
   - *Desert locust_GeneratedReport.pdf*
2. Comparison raw Data
   - *Honeybee_ComparisonRawData.txt*
   - *Damselfly_ComparisonRawData.txt*
   - *Dragonfly_ComparisonRawData.txt*
   - *Desert locust_ComparisonRawData.txt*
3. Comparison tables
   - *Honeybee_ComparisonTable.csv*
   - *Damselfly_ComparisonTable.csv*
   - *Dragonfly_ComparisonTable.csv*
   - *Desert locust_ComparisonTable.csv*
4. All pairs' results
   - *Wing Analogy, all pairs' result.pdf*
5. All pairs' sets' results
   - *Wing Analogy, all pairs' sets' Result.pdf*
